# Modified Taylor Impact Tests with Profiled Copper Cylinders: Experiment and Optimization of Dislocation Plasticity Model

**DOI:** 10.3390/ma16165602

**Published:** 2023-08-12

**Authors:** Egor S. Rodionov, Victor V. Pogorelko, Victor G. Lupanov, Polina N. Mayer, Alexander E. Mayer

**Affiliations:** Department of General and Theoretical Physics, Chelyabinsk State University, 454001 Chelyabinsk, Russia; rodionoves.pgd@gmail.com (E.S.R.); vik_ko83@mail.ru (V.V.P.); victr@csu.ru (V.G.L.); polina.nik@mail.ru (P.N.M.)

**Keywords:** Taylor impact test, profiled cylinders, SPH, dislocation plasticity, artificial neural network, Bayesian calibration

## Abstract

Current progress in numerical simulations and machine learning allows one to apply complex loading conditions for the identification of parameters in plasticity models. This possibility expands the spectrum of examined deformed states and makes the identified model more consistent with engineering practice. A combined experimental-numerical approach to identify the model parameters and study the dynamic plasticity of metals is developed and applied to the case of cold-rolled OFHC copper. In the experimental part, profiled projectiles (reduced cylinders or cones in the head part) are proposed for the Taylor impact problem for the first time for material characterization. These projectiles allow us to reach large plastic deformations with true strains up to 1.3 at strain rates up to 10^5^ s^−1^ at impact velocities below 130 m/s. The experimental results are used for the optimization of parameters of the dislocation plasticity model implemented in 3D with the numerical scheme of smoothed particle hydrodynamics (SPH). A Bayesian statistical method in combination with a trained artificial neural network as an SPH emulator is applied to optimize the parameters of the dislocation plasticity model. It is shown that classical Taylor cylinders are not enough for a univocal selection of the model parameters, while the profiled cylinders provide better optimization even if used separately. The combination of different shapes and an increase in the number of experiments increase the quality of optimization. The optimized numerical model is successfully validated by the experimental data about the shock wave profiles in flyer plate experiments from the literature. In total, a cheap, simple, but efficient route for optimizing a dynamic plasticity model is proposed. The dislocation plasticity model is extended to estimate grain refinement and volume fractions of weakened areas in comparison with experimental observations.

## 1. Introduction

The development of various engineering and defense applications requires knowledge of the dynamic strength of metals and their connection with the plasticity mechanisms governing the mechanical response of materials. The generation of plane shock waves in metals provides well-controlled conditions of dynamic loading to experimentally measure the dynamic yield and spall strengths [1,2] by interpreting the free surface velocity histories recorded with laser interferometry. Different shock wave generators allow researchers to cover a wide range of strain rates in such experiments. Flyer plates [3,4,5,6] provide high-velocity impact and shock wave loading with strain rates ranging from 10^4^ s^−^^1^ to 10^6^ s^−^^1^ depending on the sample thickness. Strain rates of about 10^6^ s^−^^1^ and higher can be reached in the shock waves generated by high-current electron irradiation [7,8] or powerful ion irradiation [9,10]. Intensive laser pulses with short [11,12] and ultrashort [13,14,15] durations can generate in thin samples steep shock wave fronts with strain rates up to 10^7^–10^9^ s^−^^1^. In spite of the achievable high strain rates, the plane shock waves cause relatively small strains of about 0.1. Another widely used technique, the split Hopkinson pressure bar (Kolsky bar) [16,17,18], provides moderate strain rates, up to about 10^3^–10^4^ s^−^^1^, but also implies restricted strains. The simultaneous action of high strain rates and large strains is relevant for a number of problems, including dynamic compaction of metal powders, dynamic impact, and penetration.

Classical Taylor impact tests proposed in [19,20,21] imply an axisymmetric collision of a cylindrical sample with a rigid anvil with impact velocities of several hundred meters per second. Such collisions simultaneously cause large strains above one and high strain rates up to 10^4^–10^5^ s^−^^1^, especially near the colliding edge of the sample. The disadvantage of this technique in terms of interpretation of the results is connected with the highly non-uniform deformation of material through the sample. In spite of this feature, a simple approximate method to estimate the dynamic strength was proposed in the mentioned pioneering works. This estimation method was further revisited and improved by a number of authors. Nowadays, Taylor tests are used to study the properties of different materials, including pure metals [22,23,24], conventional alloys, and high-entropy [25] alloys. Various modifications of the classical scheme of experiment were proposed and used, such as the symmetric (rod-on-rod) Taylor test [26]. In addition to the direct estimation of the dynamic strength, the Taylor tests are widely used for verification and parameterization of the dynamic plasticity models [27,28,29].

In the experimental part of the present work, we perform modified Taylor tests with various sample shapes cut from a conventional symmetrical cylinder and compare the final shape of the samples with simulation predictions to test and parameterize the dislocation plasticity model [30,31] numerically implemented in a 3D case [23]. For a more complete verification of a plasticity model and a clearer definition of its parameters, a greater variety of different tests and sample shapes can be advantageous. Therefore, in this paper, we propose several modifications of the sample shape in addition to the classic Taylor tests and apply machine-learning-based optimization of model parameters. Whereas the simplest stress or strain state, such as a plain shock wave, is preferable for the analytical interpretation of deformation conditions, a complex and not uniform stress state is quite acceptable and even beneficial in the case of the application of a numerical analysis. The shape of the samples is selected in such a way as to increase the deformation of their head parts at moderate impact velocities and provide a more severe dynamic deformation process. With these profiled head parts of the samples, strain rates up to 10^5^ s^−^^1^ and true strains up to about 1 are reached at impact velocities not exceeding 130 m/s, which makes our experimental setup easier and cheaper. It should be mentioned that the dislocation model with the coefficients selected in [23] for the classical Taylor test failed to describe the results of the modified Taylor tests presented here. This fact confirms the importance of additional tests and motivates us to develop the presented automated algorithm for parameter identification.

The use of high-speed cameras together with either laser interferometery [32,33] or Hopkinson pressure bars [34] allowed the experimenters to gain more information about wave processes and deformation history from a single sort and to decrease the number of Taylor tests required for the determination of the constitutive parameters of material [35]. At the same time, comparison of the final shape of the sample remains a widely used approach [29,36]. The experimental setup for registration of the final shape without high-speed cameras is cheaper and easier to construct, while involving multiple experiments instead of a few can be beneficial due to the statistical elimination of the microstructural features of specific samples. Following this logic, we employ 50 experiments with different sample shapes and impact velocities, which give us statistically meaningful information about the dynamic response of material to parameterize the dislocation plasticity model.

Various plasticity models are developed and used in the literature to predict the behavior of metals under dynamic loading, such as the classical Zerilli-Armstrong [37] and Johnson-Cook [38] ones. Physically-based plasticity models with explicit descriptions of the dislocation ensemble are more precise in reproducing the structure of elastic-plastic shock waves in metals, as previously shown in [30,31,39,40,41,42]. The dislocation multiplication term proposed in [30] on the basis of energy-wise consideration leads to a stress-dependent multiplication rate of dislocations [43,44], which is efficient for the description of the plastic relaxation on the shock wave front, including elastic precursors [42,43]. The introduction of dislocation immobilization kinetics [31] allows one to describe the gradual increase in yield strength behind the shock front, leading to a smooth structure of release waves following the shock wave [31,45,46]. A reduced version of this model was proposed in [47], where the Orowan equation for the plastic strain rate was replaced by a modified Maxwell relaxation model with the relaxation time dependent on the density of mobile dislocations [48]. The developed model was successfully applied for the simulation of classical Taylor tests in 2D [44] and 3D [23] cases, as well as for the investigation of instability in plastic flow as a mechanism of its localization [49]. Thus, this dislocation plasticity model is one of the most promising approaches to describing dynamic deformation in a wide range of conditions, and it is used in the present research.

Parameterization of mechanical models is an ongoing challenge [50,51]. It can be performed by comparison with data from experiments or atomistic simulations, and the development of machine learning approaches is highly relevant in this field. A powerful tool for doing this is the Bayesian statistical method [52], consisting of the random enumeration of parameter sets and the estimation of a quasi-probability of each set according to the correspondence between the model predictions and the training data. Walters et al. [53] successfully employed the plate impact experiments and Bayesian method to calibrate the Johnson-Cook strength model; a fast emulator (Gaussian regression model) was trained to predict the hydrocode model outputs in order to substitute the model in the Bayesian algorithm and to speed up the optimization process. It should be mentioned that the Johnson-Cook model is rarely used for simulation of shock wave problems, but the Bayesian optimization allowed authors to obtain acceptable correspondents with the experimental free surface velocity profiles. Only four points on the shock wave front for each experimental shot were used to calibrate the model, while the elastic-plastic release wave was not considered. Nguyen et al. [18] applied a similar approach to calibrate a dislocation plasticity model and further used the calibrated model to numerically investigate deformation anisotropy in Taylor impact experiments with tantalum single crystals. Sjue et al. [36] developed the previous calibration approach [53] by incorporating data from four types of testing: quasistatic compression, split Hopkinson pressure bars, Taylor cylinders, and flyer plate impact experiments. In this set, Taylor cylinders provide large strains (up to 3), but restricted strain rates (10^4^ s^−^^1^), while flyer plate experiments provide two orders of magnitude higher strain rates (10^6^ s^−^^1^), but restricted strains (up to about 0.1). In this sense, an increase in strain rate together with retaining large strains by means of profiling the head part of the cylinder in the Taylor tests is of significant interest. Rivera et al. [29] used classical Taylor tests and a Bayesian algorithm to calibrate the Preston–Tonks–Wallace strength model [54] for tantalum. They constructed a surrogate model (an emulator) of finite element simulations of the Taylor impact problem by means of a Gaussian process and incorporated this surrogate model in the Bayesian calibration algorithm. Only three experimental profiles with slightly varied velocities were considered, and only four numbers (final positions of two selected material points) for each profile were compared with the surrogate model.

Previously, we had applied Bayesian calibration with the data of atomistic simulations for parameterization of mechanical models of several dynamic processes, including the dynamic compaction of nanoparticles [55] and nanoporous metals [56]. In all previous cases, the mechanical model worked quickly, and an emulator was not used. The Bayesian method reveals its efficiency in all cases considered, and here it is applied to parameterize the plasticity model using experimental data. As opposed to previous works in this field [18,29,36,53], we use data from many Taylor tests with profiled samples and employ an artificial neural network (ANN) to create an emulator of the full-scale numerical code instead of the Gaussian process. Profiled Taylor cylinders were previously experimentally examined in [57] for truncated conical, hemispherical, and truncated ogive shapes and hypothetically considered in the numerical simulations by [58] for truncated conical (Impala’s horn) shapes, but they were not used for calibration of the strength model in these papers; reduced cylinders as a head part are considered in addition to the truncated cone. Also, the smoothed particle hydrodynamics (SPH) with embedded dislocation plasticity model as proposed in [23] is used to perform 3D simulations of the impact instead of finite element modeling. The SPH was originally designed to solve astrophysical problems [59], but nowadays it is entrenched in solid mechanics as well [23,60,61]. The meshless SPH allows one to consider large strains and destruction of material without technical problems with distortion of the computational grid.

ANN is a convenient tool for approximation of data sets with complex and/or unknown functional dependence that is actively applied in the mechanics of materials [62,63,64,65]. Nowadays, ANNs are fruitfully used to generalize calculation data from both SPH [66,67,68,69] and finite element analysis [70] for the solution of various engineering problems. An ANN can be embedded in finite element [71] or finite difference [56] simulations as a fast-running part of a multiscale model. It was previously shown [72] that a complex dependence between the elastic strain and stress tensor components can be efficiently approximated by an ANN trained on molecular dynamics (MD) data and further used in continuum mechanics modeling as a tensor equation of state (EOS). A similar approach was justified in [23] to obtain a scalar EOS of copper. The scalar EOS connects density and internal energy with the pressure and temperature of the material. Typically, it is constructed in analytical [73] or tabular form, but here it is substituted by an ANN. The EOS can be parameterized by experimental data or atomistic simulation data, as well as by their combination. In our case, ANN training replaces parameterization, and only MD simulations are used to generate the training data set. In this sense, our EOS relies on the accuracy of interatomic potential in the MD. On the one hand, the MD-ANN-based EOS seems excessive for copper, for which a variety of conventional EOSs were built. On the other hand, the development of this approach has advantages when applied to complex or unexplored materials. Thus, ANNs are used for both generalization of SPH results and approximation of the EOS.

The present research continues and develops our previous work [23]. For the first time, we employ multiple Taylor tests with profiled cylinders to increase the strain and the strain rate in the head part at moderate impact velocities. Further, we employ 5 numerical characteristics of the final deformed shape of the sample for each one of 50 experiments (in total, 250 values) to verify and parameterize the dislocation plasticity model [31] by means of a machine-learning-type Bayesian algorithm. An ANN is trained and used as a model emulator to speed up Bayesian parameterization. In total, our work identified a cheap, efficient, and statistically meaningful route to determine the experimental parameters of a plasticity model.

## 2. Materials and Methods

### 2.1. Materials, Specimens, and Gas Gun Launcher

**Figure 1:**
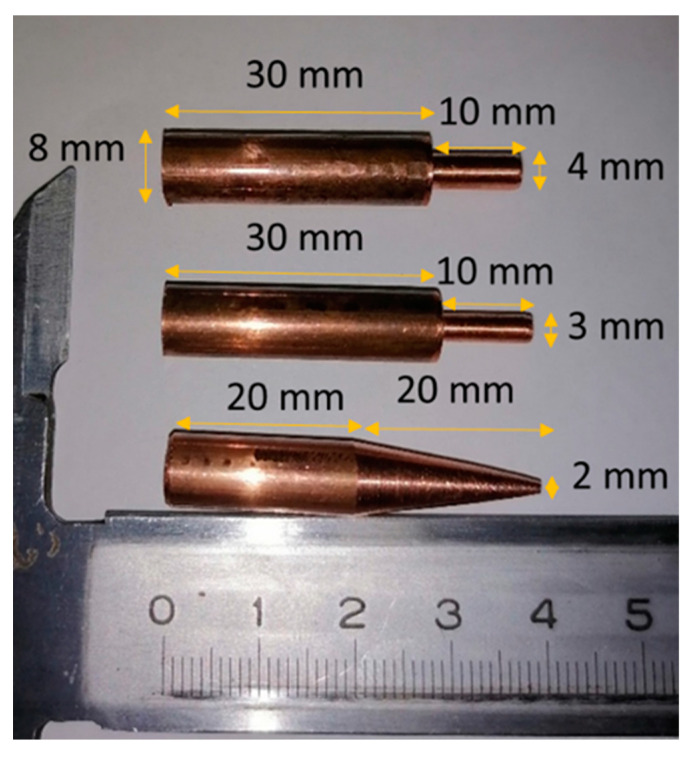
Initial shapes of the profiled copper cylinders used in the modified Taylor tests.

Cold-rolled oxygen-free copper (OFHC) is used as a flaying sample, and stainless steel is used as an anvil. The samples were made of 99.9% copper of M1 grade (similar to C11000 in the U.S. and Cu-ETP in Europe). Specification gives the following upper limits for impurities: 0.001% Bi, 0.005% Fe, 0.002% Ni, 0.004% Zn, 0.002% Sn, 0.002% Sb, 0.002% As, 0.005% Pb, and 0.004% S. OFHC is an important FCC metal with high thermal and electrical conductivity. It is extensively used in structures and machines that can be subjected to dynamic loads ranging from electric vehicles to rockets and projectiles. The as-received material was in the form of cold-rolled cylindrical rods with a diameter of 8 mm. The rods were cut on cylinders 40 mm in length and machined in order to profile the head part into the following three shapes (see Figure 1): (i) the reduced cylinder, 4 mm in diameter and 10 mm in length; (ii) a similar cylinder with a diameter of 3 mm; (iii) the truncated cone with a top diameter of 2 mm and a length of 20 mm. The profiling allows us to increase the resulting strain and strain rate at restricted impact velocity by diminishing the zone of plastic deformation at almost the same total mass and energy of the projectile. As a result, the impact energy is concentrated in a smaller part subjected to large plastic deformation. As far as we know, this profiling of samples for the Taylor test is being proposed for the first time. The mass of the copper sample is about 14.5 g for the 4-mm cylinder head, 14 g for the 3-mm cylinder head, and 12.7 g for the truncated cone head part. These experiments are supplemented by the classical Taylor tests with non-profiled cylinders [23].

**Figure 2:**
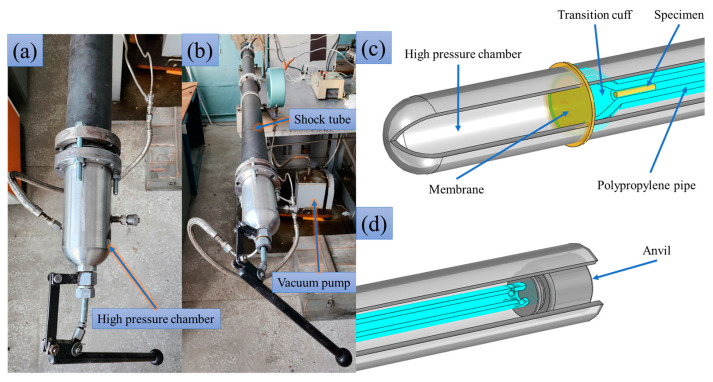
Experimental setup: (**a**) photograph of the high-pressure chamber; (**b**) photograph of the shock-wave tube and vacuum pump; (**c**) scheme of the high-pressure chamber and initial part of barrel; (**d**) scheme of the final part of barrel and anvil.

The samples collide with velocities varying from 19 to 125.2 m/s with a rigid anvil made of polished stainless steel 304. An air gas gun was constructed on the basis of the shock wave tube with a diameter of 66 mm and a length of 2.3 m, equipped with a high-pressure chamber of the same diameter and length of 0.3 m (Laboratory of Applied Gas Dynamics of the Chelyabinsk State University, Chelyabinsk, Russia); see Figure 2a,b. The schema of the setup is presented in Figure 2c,d. The accelerating channel is made of polypropylene pipe with a length of 2.1 m and an internal diameter of 12 mm installed inside the shock wave tube in order to make the accelerating channel diameter closer to the sample diameter. A uniform inflow of compressed air from the high-pressure chamber into the polypropylene pipe is provided by a cone-shaped transition cuff. The impact velocity is controlled by the injected pressure in the high-pressure chamber; the maximum pressure used is 10 bar. The pressure in the accelerating channel is reduced to about 0.05 bar by the vacuum pump. The separating diaphragm is made of polypropylene tape glued together in several layers. A fluoroplastic shell with a low friction coefficient and a mass of about 5 g is used to tightly fit the sample in the cannel. WD-40 is also applied to the shell and the open parts of the sample in order to reduce friction. The time-of-flight method is used to measure the projectile velocity near the anvil, as described in detail in [23].

### 2.2. Dislocation Plasticity Model

A continuum model of dislocation plasticity is used, which was first proposed in [30] and further improved in [31] by introducing more detailed dislocation kinetics, taking into account immobilized dislocations and stronger hardening. Previously, the model was numerically implemented in the 1D [30,31,43,74] and 2D [7,44] cases using a finite difference scheme, as well as in the 3D case [23] using the SPH (smoothed particle hydrodynamics) approach. For the sake of completeness, the dislocation plasticity model is formulated here based on previous publications [23,31,74].

Conservation laws form a core of a dynamic model of continuum mechanics [75] and determine the evolution of density ρ, particle velocity v, and the specific internal energy $E_{\Sigma }$, which can be represented as a sum $E_{\Sigma }=E+E_{S}+E_{D}$, where E is the part associated with hydrostatic compression and heating, $E_{S}$ is the part associated with elastic change in shape, and $E_{D}$ is the energy of lattice defects (dislocations). Using the Lagrangian frame of reference, the conservation laws can be written as follows:

(1) $$\frac{d\rho }{dt}=-\rho (\nabla \cdot v),$$

(2) $$\rho \frac{dv}{dt}=(\nabla \cdot \sigma ),$$

(3) $$\rho \frac{dE}{dt}=-P\left(\nabla \cdot v\right)+\beta (S:\dot{w}),$$

where σ=−PI+S is the stress tensor, P is the pressure, S is the tensor of stress deviators, and I is the identity tensor. The last term on the right-hand side of Equation (3) is the heating due to plastically dissipated mechanical energy, where $\dot{w}$ is the plastic strain rate, and β is the Taylor–Quinney coefficient [76]; it is assumed that β=0.9 in the present study. As discussed in [74], the work of the stress deviators is first accumulated in $E_{S}$ and then transferred to both E and $E_{D}$ by means of plastic dissipation.

Stresses σ=−PI+S must be calculated in order to apply Equations (2) and (3). Pressure P and temperature *T* can be expressed in terms of the equation of state knowing two other thermodynamic parameters, the density ρ and the “hydrostatic” part *E* of internal energy in our case. For this purpose, an equation of state in the form of an artificial neural network (ANN) is used, as described in Section 2.4. The stress deviator S is determined by elastic-plastic behavior. The elastic-plastic state is described by the macroscopic strain tensor u and the plastic strain tensor w. In the case of small elastic deformations, one can use the difference u−w as the elastic strain tensor and apply Hooke’s law [77]:

(4) $$S=2G[u-\frac{1}{3}\mathrm{tr}\left(u\right)I-w],$$

where G is the shear modulus. The evolution of the macroscopic strain tensor is determined as follows:

(5) $$\frac{du}{dt}=\frac{1}{2}\left[\left(\nabla ⊗v\right)+{\left(\nabla ⊗v\right)}^{T}\right]+[\left(u\cdot \dot{R}\right)+\left({\dot{R}}^{T}\cdot u\right)],$$

where the superscript “T” stands for transposition and “⊗” means dyadic product. The first term on the right-hand side of Equation (5) is the strain rate, and the second term takes into account the rotation of substance elements with the rotation rate tensor:

(6) $$\dot{R}=\frac{1}{2}[\left(\nabla ⊗v\right)-{\left(\nabla ⊗v\right)}^{T}].$$

The change of w is determined by both the plastic strain rate $\dot{w}$ and rotation:

(7) $$\frac{dw}{dt}=\dot{w}+[\left(w\cdot \dot{R}\right)+\left({\dot{R}}^{T}\cdot w\right)].$$

Plasticity is provided by the dislocation slip and is described by the Orowan equation:

(8) $$\dot{w}=\sum_{\delta }bM^{\delta }\rho_{D}^{\delta }V_{D}^{\delta },$$

where δ∈[1,12] enumerates slip systems of the FCC crystal [78] and is represented by four slip planes equivalent to (111) one and three Burgers vectors in each plane equivalent to [110]; b is the modulus of the Burgers vector; $\rho_{D}^{\delta }$ is the scalar density of the mobile dislocations in the corresponding slip system, and $V_{D}^{\delta }$ is the slip velocity of the dislocations in the slip system. The orientation tensor $M^{\delta }$ is defined as the symmetric part of the binary product of the Burgers vector direction $b^{\delta }/b$ and the normal to the slip plane $n^{\delta }$:

(9) $$M^{\delta }=\frac{1}{2b}[\left(b^{\delta }⊗n^{\delta }\right)+{\left(b^{\delta }⊗n^{\delta }\right)}^{T}].$$

The change in the orientation tensor in time is determined by the rotation of substance elements:

(10) $$\frac{dM^{\delta }}{dt}=[\left(M^{\delta }\cdot \dot{R}\right)+\left({\dot{R}}^{T}\cdot M^{\delta }\right)].$$

The initial orientation of the slip system is the same through a single crystal, while it varies between grains in polycrystalline copper. In order to mimic a polycrystalline sample, different initial orientations in each call of the numerical grid are randomly seeded, as proposed in [30].

The Peach-Koehler force [77,79] per unit length of dislocation acting on dislocations of the *δ*-th slip system in the slip direction is equal to the following:

(11) $$F^{\delta }=b(M^{\delta }:S).$$

The inertia of dislocations is very small: the balancing time is about 10^−11^ s [80], which is much smaller than the time step for macroscopic modeling. Therefore, a quasi-stationary solution [74,80] is used for the dislocation velocity:

(12) $$V_{D}^{\delta }=\frac{c_{t}\zeta^{\delta }}{6\sqrt{6}\chi^{\delta }}{\left[{\left(\chi^{\delta }\right)}^{2/3}-12\right]}^{3/2},$$

(13) $$\zeta^{\delta }=\frac{1}{c_{t}B}\left(F^{\delta }-\frac{bY_{s}}{2}\mathrm{sign}\left(F^{\delta }\right)\right)\cdot H(\left|F^{\delta }\right|-\frac{bY_{s}}{2}),$$

(14) $$\chi^{\delta }=108\left|\zeta^{\delta }\right|+12\sqrt{3}\sqrt{4+27{\left|\zeta^{\delta }\right|}^{2}},$$

where H(·) is the Heaviside step function; $c_{t}=\sqrt{G/\rho }$ is the transverse sound speed; and $Y_{s}$ is the static yield stress. The friction coefficient B at room temperature is taken from the MD simulation [81] for copper, while the temperature dependence is assumed to be the same as for aluminum [80]; see Table 1.

The kinetics of dislocations takes into account both mobile and immobilized dislocations, characterized by the scalar densities $\rho_{D}^{\delta }$ and $\rho_{I}^{\delta }$, respectively. The kinetics equations express the balance between the rates of multiplication $Q_{D}^{\delta }$, immobilization $Q_{I}^{\delta }$, and annihilation of the pairs of mobile-mobile $Q_{A}^{\delta }$ and mobile-immobilized $Q_{\mathrm{AI}}^{\delta }$ dislocations [31]:

(15) $$\frac{d\rho_{D}^{\delta }}{dt}=Q_{D}^{\delta }-Q_{I}^{\delta }-Q_{A}^{\delta }-Q_{\mathrm{AI}}^{\delta }-\rho_{D}^{\delta }(\nabla \cdot v),$$

(16) $$\frac{d\rho_{I}^{\delta }}{dt}=Q_{I}^{\delta }-Q_{\mathrm{AI}}^{\delta }-\rho_{I}^{\delta }(\nabla \cdot v).$$

An energy-based approach [30] is used for calculating the dislocation multiplication rate:

(17) $$Q_{D}^{\delta }=k_{D}b\rho_{D}^{\delta }\left|F_{D}^{\delta }V_{D}^{\delta }\right|,$$

where the generation coefficient $k_{D}=\left(1-\beta \right)/\left(8\;\mathrm{eV}\right)$ is part (1−β) of the power of the plastic dissipation $\rho_{D}^{\delta }\left|F_{D}^{\delta }V_{D}^{\delta }\right|$ spending on the formation of new defects divided by the energy of dislocation formation per length of one Burgers vector equal to about 8 eV. The immobilization rate can be written as follows [31]:

(18) $$Q_{I}^{\delta }=V_{I}\left(\rho_{D}^{\delta }-\rho_{D}^{\mathrm{free}}\right)\sqrt{\rho_{I}^{\delta }},$$

where $\rho_{D}^{\mathrm{free}}$ is a threshold value of dislocation density starting the immobilization. Equation (18) describes the formation of strong dislocation structures with a constant velocity $V_{I}$ of dislocation motion during this process.

The annihilation rates are written in a common way:

(19) $$Q_{A}^{\delta }=2k_{A}b\left|V_{D}^{\delta }\right|{\left(\rho_{D}^{\delta }\right)}^{2},\;\;\;\;Q_{\mathrm{AI}}^{\delta }=k_{A}b\left|V_{D}^{\delta }\right|(\rho_{D}^{\delta }\rho_{I}^{\delta }),$$

where $k_{A}$ is the annihilation coefficient. The initial values of the mobile density in each slip system and the parameters of the kinetics model are collected in Table 1.

The examined cold-rolled copper initially has a high dislocation density, which is additionally increased during the dynamic tests. Therefore, strain hardening is an important factor determining the plastic flow during the Taylor tests. The strain hardening is taken into account according to the Taylor hardening law:

(20) $$Y_{s}=Y_{s0}+A_{I}Gb\sqrt{\rho_{I}},\;\;\;\rho_{I}=\sum_{\delta }\rho_{I}^{\delta },$$

where $Y_{s0}$ is the static yield stress in the material without dislocation. Equation (20) considers only the immobilized dislocations, but the value of the hardening coefficient $A_{I}$ excides the commonly used one. The high value of $A_{I}$ is attributed to dislocation structures consisting of immobilized dislocations and creating strong obstacles for the motion of mobile dislocations. Such a strong hardening by immobilized dislocations makes it possible to describe the structure of the unloading wave propagating behind the shock wave in plate impact experiments [31,45,46]. Isotropic hardening is taken into account in Equation (20); a more detailed description of single crystals can be achieved by taking into account anisotropic hardening [18,82,83], but it is excessive for polycrystals.

Parameters of the dislocation plasticity model are collected in Table 1. Most of them are taken from the previous works, while several parameters, including the hardening coefficient $A_{I}$, the immobilization velocity $V_{I}$, and the initial density of immobilized dislocations $\rho_{I}^{\delta }\left(t=0\right)$, are fitted to the present experiments. We show that the selection of these three parameters by the statistical Bayesian method makes it possible to adequately describe the deformations of all considered specimen shapes.

### 2.3. Grain Refinement and Weakened Areas (Pore-like Structures)

The dislocation plasticity model [31] reduced the modification of material microstructure to changes in densities of mobile and immobilized dislocations (Equations (15) and (16)), while a number of specific microstructures can form in real materials due to plastic deformation. Grain refinement and the formation of pore-like structures are observed by means of optical microscopy for both classical [23] and profiled (Section 3.2) cylinders; here we extend the dislocation plasticity model to estimate these effects.

The energy-wise consideration proposed in [30,31] for dislocation multiplication revealed itself as an efficient approach for dynamic problems. According to Equation (17), a part of the plastic work is transferred to the dislocation subsystem, but dislocations cannot accumulate this work endlessly. The dislocation density saturates due to annihilation (Equation (19)), and the total power released in unit volume due to the annihilation can be calculated as follows:

(21) $$\Pi =\varepsilon_{D}\sum_{\delta }\left(Q_{A}^{\delta }+2Q_{\mathrm{AI}}^{\delta }\right),$$

where $\varepsilon_{D}=8\;\mathrm{eV}/b$ is the dislocation energy per unit length of dislocation line. In the model, the annihilation terms express all effects of dislocation density decrease, which, in real material, include absorption by grain boundaries (GBs) leading to the misorientation angle increase and formation of high-angle grain boundaries. Thus, one can conclude that a part $\eta_{\mathrm{GB}}$ of the released power, Equation (21), is spent on the formation of high-angle GBs, it means, on the grain refinement. One can estimate GB surface area per unit volume as the ratio of cube surface to the cube volume divided by 2 (because each boundary belongs to two grains): 3/d, where d is the grain size (diameter); this is an estimate from above, because real grains are rounded and have a smaller specific area of GBs. The grain boundary energy complexly depends on the misorientation and has certain minimums for specific misorientations [84]; besides it, this energy is increased for metastable states of GB [85]. Nevertheless, the energy of grain boundaries of common type in copper can be estimated at the level of $\gamma_{\mathrm{GB}}=0.5\;{J/m}^{2}$ [84]. Thus, the local grain refinement to the moment t can be estimated as follows:

(22) $$d={\left[d_{0}^{-1}+\frac{\eta_{\mathrm{GB}}}{3\gamma_{\mathrm{GB}}}\int_{0}^{t}\Pi \;dt^{\prime }\right]}^{-1},$$

where $d_{0}$ is the initial grain diameter before the dynamic loading $d_{0}=d\left(t=0\right)\approx 18\;\mu m$.

The formation of pore-like structures is observed in our experiments besides grain refinement. The remaining part $\left(1-\eta_{\mathrm{GB}}\right)\Pi$ of the released power is assumed to be spent on the complete break of atomic bonds of a part of atoms. The fraction of atoms with broken bonds can be estimated from below as follows:

(23) $$f=\frac{\left(1-\eta_{\mathrm{GB}}\right)}{\varepsilon_{S}n_{0}}\int_{0}^{t}\Pi \;dt^{\prime },$$

where $n_{0}∼10^{29}\;m^{-1}$ is the concentration of atoms and $\varepsilon_{S}\approx Gb^{3}\approx 4.5\;\mathrm{eV}$ is the sublimation energy. The atomistic mechanism of such energy transformation can consist of the formation of vacancies and defect clusters during the annihilation of dislocations observed in MD studies [86,87]. We do not consider that the observed pore-like structures are real pores formed by vacancies, “sublimated atoms”, or due to the plastic growth of pores under negative pressure, because the calculated stress evolution does not reveal sufficiently high negative pressures in the sample. The pore-like structures are rather weak areas with disordered surfaces that are chipped out during the section etching before the microscopy. Assuming that the atomic bonds are completely broken only in the surface area of the thickness b around each pore-like structure, the volume fraction of these weakened areas is estimated as follows:

(24) $$\alpha =\frac{\left(\pi D^{3}/6\right)}{\left(\pi D^{2}b\right)}f=\frac{D\left(1-\eta_{\mathrm{GB}}\right)}{6b\varepsilon_{S}n_{0}}\int_{0}^{t}\Pi \;dt^{\prime },$$

where D is diameter of pore-like structures. Optical microscopy gives diameters ranging from 10 to 40 µm, and D=20 μm is used as an estimate.

The obtained Equations (22) and (24) allow us to estimate the effect of grain refinement and the formation of weakened areas in the frames of the dislocation plasticity model in comparison with the experimental data. Concerning the energy distribution between these two processes, we simply assume $\eta_{\mathrm{GB}}=0.5$. Equations (23) and (24) can be directly used for the development of the model of plasticity-stimulated fracture of metallic materials.

### 2.4. Equation of State in the Form of MD-Informed ANN

The hydrostatic part of internal energy E and density ρ determine the pressure, bulk modulus K, and the temperature T by means of the equation of state:

(25) $$P=P_{\mathrm{EOS}}\left(\rho ,\;E\right),\;\;\;\;K=K_{\mathrm{EOS}}\left(\rho ,\;E\right),\;\;\;\;T=T_{\mathrm{EOS}}\left(\rho ,\;E\right),$$

while the shear modulus G can be expressed in terms of the bulk modulus K and Poisson’s ratio μ [31]:

(26) $$G=\frac{2}{3}K\frac{1-2\mu }{1+\mu }.$$

The equation of state is constructed in the form of an artificial neural network (ANN) trained according to MD data for hydrostatic compression and tension of a representative volume of copper elements, as described in detail in [23]. Although pressure and shear stresses are interconnected in the case of the nonlinear elastic behavior of perfect single crystals [72], the independence between pressure and shear stress is commonly postulated in the modeling of shock waves and dynamic impact due to the fact that the elastic strains remain small. An ANN-based tensor equation of state is constructed in [72] for limited cases of deformation. In the case of arbitrary 3D deformation, this procedure requires much more MD simulations and remains in perspective. Therefore, a scalar equation of state is used in the present study.

An equation of state maps the input vector {ρ; E} onto output vector {P; T; K}. An ANN is an efficient procedure for approximating a complex relationship without a predefined functional form [88,89]. For the preparation of the training data, the hydrostatic compression of copper up to 100 GPa and its tension up to fracture were investigated using MD simulations for temperatures in the range from 100 K to 900 K. Deformation was applied at a constant engineering strain rate of 10^9^ ns^−1^ under a constant temperature supported by a Nose-Hoover thermostat [90]. The MD system contained half a million atoms. The simulation was performed using the LAMMPS package [91] and the force field [92], which are widely used and verified for Cu, Al, and Al-Cu systems. The total kinetic energy and virial theorem were used to calculate the average pressure [93], and the calculated stress-strain and energy-strain curves were used for ANN training. The bulk modulus was found as the slope of the pressure-volumetric strain curve determined by the least squares method. MD data processing allowed the preparation of pairs of input {ρ; E} and output {P; T; K} vectors; in total, the training data set contained 2550 pairs. A more detailed description of the structure and training of the ANN is presented in [23].

### 2.5. Numerical Implementation

The numerical solution is implemented as the FORTRAN program SPHEP (SPH for Elastic-Plastic flows) described in our previous paper [23]. The SPH approach supposes the division of a continuum medium on particles with mechanical characteristics smudged by using a smoothing kernel [94]. The calculation of spatial derivatives is reduced to summation over neighboring particles. In order to facilitate this summation, localized kernels are usually used, and we use $M_{4}$ kernel [94] based on a cubic spline. This kernel is zero at distances larger than 2h, where h is the smoothing scale. The time of summation determines the performance of the algorithm to the greatest extent. For compiling a list of neighboring particles, the computation domain is divided on rectangular cells with sides not less than 2h, and each particle is assigned to a cell. Only particles in 3 × 3 × 3 rectangles closest to the current particle can contribute to the spatial derivatives calculated at the current particle.

The SPH particles are initially placed in the sites of the body-centered cubic (bcc) lattice, which is used for the arrangement of SPH particles as discrete representatives of the medium. This arrangement provides a better result, including better connections between SPH particles, than a simple cubic one. The linear scale of smoothing h is chosen to be twice the initial distance between the particles along the edges of the cube. Each calculation is performed in a one-thread mode, but optimization in computation time is achieved by running a set of calculations (several tens of threads) for different impact velocities, sample shapes, and, probably, model parameters. Other details of the implementation of the SPH are described elsewhere [23].

**Figure 3:**
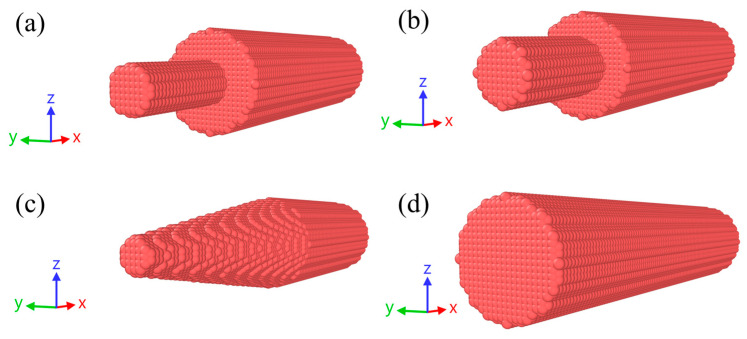
The shape of the samples before deformation in a numerical experiment. (**a**) For a 3 mm reduced cylinder, the number of SPH particles is about 50,000; (**b**) For a 4 mm reduced cylinder, the number of SPH particles is about 51,000; (**c**) For a cone with a head part diameter of 2 mm, the number of SPH particles is about 45,000; (**d**) For an 8 mm cylinder, the number of SPH particles is about 63,000.

The numerical models used for 3D calculations of the plasticity of dislocations are presented in Figure 3. The OVITO program [95] was used to visualize the obtained results. The number of SPH particles was determined from preliminary parametric studies so that there is a balance between accuracy and computation time. Free boundary conditions were set on all surfaces of the projectile, except for the contact with the anvil, which is impermeable and free-sliding. All SPH particles are given a uniform initial velocity towards the anvil. 

**Figure 4:**
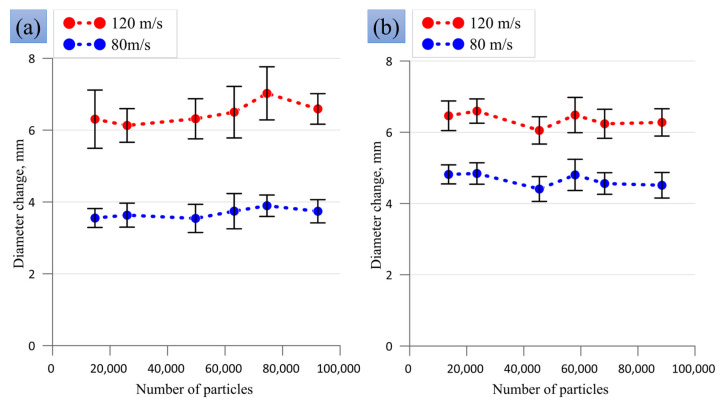
Influence of the number of SPH particles on the change in the diameter of the head part of the projectile for (**a**) a 3 mm reduced cylinder and (**b**) a truncated cone. Blue shows the curve at an impact speed of 80 m/s; red shows 120 m/s.

Figure 4 shows the effect of the number of SPH particles in the numerical models on the change in the diameter of the sample head part for cases of a reduced 3 mm cylinder and a truncated cone. These shapes are indicative because they contain the smallest number of SPH particles, especially in the head part, in comparison with other sample shapes, which can affect the computing accuracy. An increase or decrease in the number of particles shown in Figure 3 does not significantly affect the change in diameter in the numerical experiment. The average values vary nonmonotonically with the number of SPH particles, and this variation is within the error ranges. The error ranges shown in Figure 3 result from the lack of exact axial symmetry in the numerical model, which leads to different changes in diameter along different longitudinal sections. We chose an optimal value of SPH particle density, leading to about 50,000 particles per sample, as shown in Figure 3, which allows a good description of the length and diameter of the samples, and relatively fast calculations. A further increase in the number of particles increases the calculation time, while the accuracy remains the same.

Friction between sample and anvil is not taken into account in the main calculations. The parametric study presented in Appendix C shows that the deformed shape contradicts the experimental one for friction coefficients of 0.05 and higher. This fact can be explained by applying WD-40 to reduce the friction in experiments, as described in Section 2.1, as well as by a nonlinear dependence between the friction force and the normal force, effectively reducing the friction coefficient at high normal forces.

## 3. Results

### 3.1. Results of Experiments

**Figure 5:**
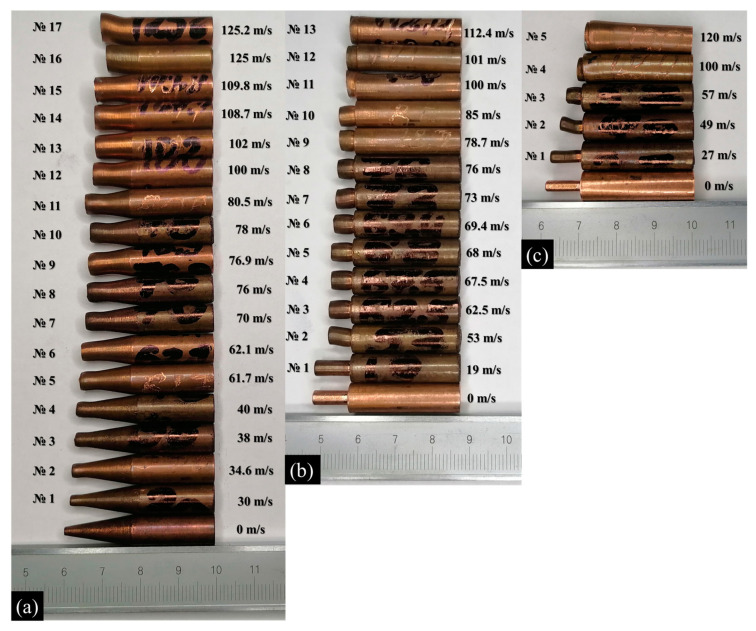
Final samples at different impact velocities; initial samples are the cylinders with the following head parts: (**a**) cone, (**b**) reduced 4-mm cylinder, and (**c**) reduced 3-mm cylinder.

Figure 5 shows the final shape of impacted specimens in comparison with the reference sample for the three considered profiles of the head part. Some of the impacted samples are not shown in Figure 5 because they were cut for microstructural analysis. In the case of the cylindrical head part shown in Figure 5b,c, the main trend of deformation is the shortening of the head part. The degree of shortening monotonously increases with the increase in impact velocity. A lower diameter of the head part leads to stronger shortening at the same impact velocity. Radial expansion is close to uniform along the head part. The radial true strain calculated from the diameter of the head part reaches about 0.7 for the 4-mm head part and about 1 for the 3-mm head part at maximal impact velocities in the experiment. The tail cylinder remains almost undeformed up to impact velocities of about 100 m/s. This tail cylinder works as a hammer, striking and compressing the head part of the samples. For experiments with a reduced cylinder with a high impact velocity above 100 m/s, one can observe the deformation of the tail part of the cylinder (samples No. 11–13 for the 4-mm reduced cylinder, and samples No. 4 and 5 for the 3-mm reduced cylinder) in Figure 5b,c.

In the case of the conical head part (Figure 5a), the deformation is substantially non-uniform along the axis, which is typical for the classical Taylor tests. In contrast to the “mushrooming” of the classical cylindrical samples, the increased deformation at the end of the cone leads to some leveling of the diameter of the deformed specimen near the impacted edge. The maximal radial true strain at the cone tip reaches about 1.3 at an impact velocity above 100 m/s. The shortening of the cone part is obvious in Figure 5a.

### 3.2. Optical Metallographic Analysis

**Figure 6:**
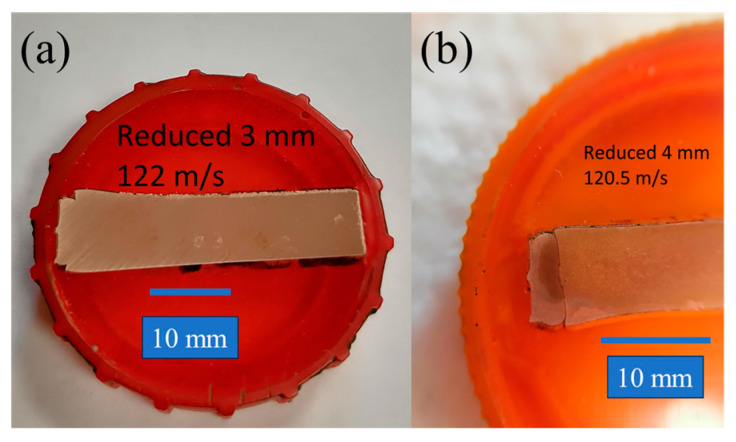
Preparation for metallographic analysis: (**a**) reduced 3-mm cylinder after polishing and before etching, the impact velocity is 122 m/s; (**b**) reduced 4 mm cylinder after etching. The impact velocity is 120.5 m/s.

Metallographic analysis by means of optical microscopy with a maximal magnification of 500 times is performed for the following samples: The 3-mm reduced cylinders impacted at 89.3, 100, 120.5, and 122 m/s; the 4-mm reduced cylinders impacted at 73 and 120.5 m/s; and the cone impacted at 120.5 m/s. The main goal was to investigate the initial microstructure and its change during the dynamic deformation. The preparation of cross-sections was worked out in [23]: the samples were placed in a container, fixed by epoxy resin, and subjected to manual micro-polishing using moisture-resistant sandpaper with periodic cooling in water. A 3% solution of hydrogen peroxide, citric acid, and salt (sodium chloride) was used for etching. Photos of polished (a) and etched (b) samples are shown in Figure 6. The grain diameter is estimated by the area method.

**Figure 7:**
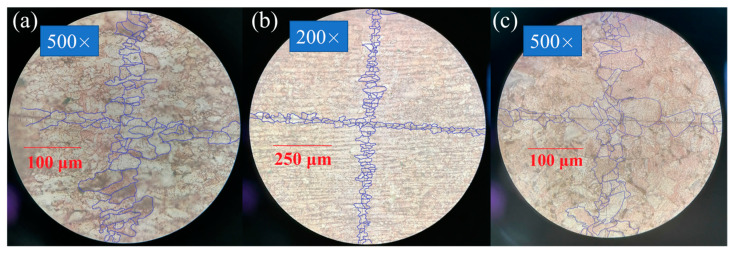
Cross-sectional optical micrographs of the as-received copper sample obtained using a metallographic microscope; (**a**,**b**) grains are marked for size estimation; (**c**) the subgrain structure. Subgrains are smaller than 3 µm.

**Figure 8:**
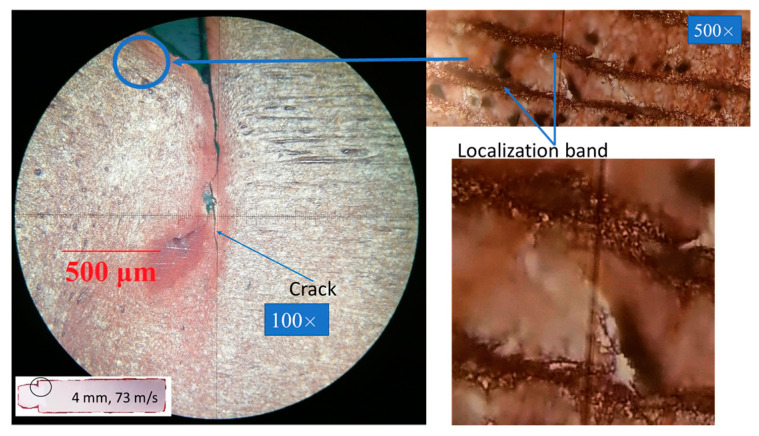
Cross-sectional optical micrographs of a deformed 4-mm reduced sample impacted with a velocity of 73 m/s: visible cracks in the area of the transition diameter of the cylinder and localization bands of plastic flow with a characteristic size of 10–20 µm.

At first, the as-received samples are considered, whose microstructure originates from the quasi-static deformation during the cold rolling used at the stage of copper rod manufacturing. No impurities or oxides are observed in the micrographs. The grains with an average grain diameter of about 18 ± 4 µm are elongated in the rolling direction, as shown in Figure 7a–c. A mesh structure of subgrains with a size of up to 3 µm can be seen in Figure 7c. Localization bands of plastic flow with a width of 10–20 µm are clearly visible. These strips go along the direction of rolling of the copper rod and look like small depressions consisting mainly of small grains less than 3 µm in size, as shown in Figure 8. It is well known from the literature that up to 90% of the entire plastic deformation of the material can be concentrated in shear bands, whose formation is of great interest [96,97,98,99,100]. Shear bands have a complex structure of strongly deformed material and contain structures of dislocations and nanocrystalline grains [101,102,103,104,105]. Subgrain boundaries and shear bands form strong dislocation structures with a large density of immobilized dislocations, which hinder the slip of movable dislocations and increase the yield strength. Let us estimate the density of dislocations creating subgrain boundaries if the latter can be considered dislocation walls (low-angle grain boundaries). If one estimates the average diameter of subgrains as $D_{\mathrm{SG}}\;=\;3\;\mu m$, the specific area of subgrain boundaries can be estimated as half of the ratio of the sphere surface area to the sphere volume: $3/D_{\mathrm{SG}}=10^{6}m^{-1}$. The planar density of dislocations in low-angle grain boundaries varies from about $10^{8}\;m^{-1}$ to about $8\times 10^{8}\;m^{-1}$ at the misorientation angle, which increases from 2° to 15° [106]. Thus, one can estimate the scalar (volumetric) density of dislocations forming these structures in the range from $1\times 10^{14}\;m^{-2}$ to $8\times 10^{14}\;m^{-2}$. This estimation does not contradict the experimental data of [107], who measured by means of transmission electron microscopy the value of about $1.2\times 10^{14}\;m^{-2}$ with the formation of cell structure at 10% tensile deformation of OFHC copper at room temperature. It should be noted that the fitted value of the initial total dislocation density (sum over all slip systems) in the dislocation plasticity model is about $0.8\times 10^{14}\;m^{-2}$ (see Table 1), which is of the same order as our estimates from the optical metallography and the experimental data of [107].

**Figure 9:**
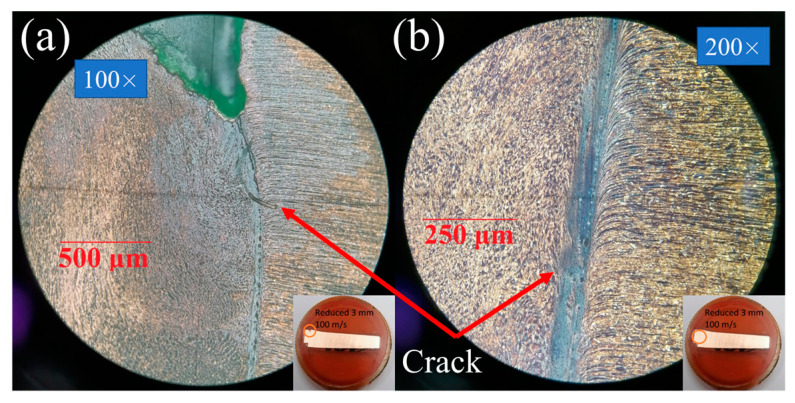
Cross-sectional optical micrographs of a deformed 3-mm reduced sample impacted with a velocity of 100 m/s: (**a**) area near the transition diameter from the head part (from the left) to the main part (from the right) with a crack; (**b**) central part of the transition diameter with a through crack.

**Figure 10:**
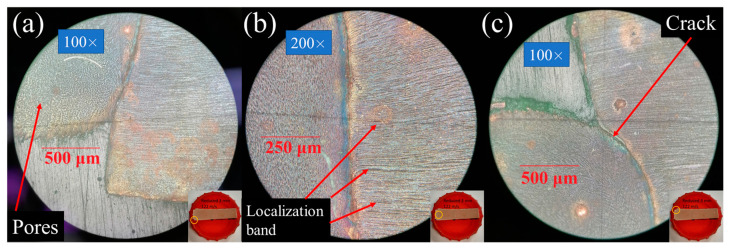
Cross-sectional optical micrographs of a deformed 3-mm reduced sample impacted with a velocity of 122 m/s: (**a**) Pores with a diameter of 10–40 μm are visualized in the deformed head part of the sample; (**b**,**c**) pores are observed in the region of the transition diameter of the sample to the left of the crack, and localization bands are observed near the crack.

Figure 8, Figure 9 and Figure 10 show the microstructure of the dynamically deformed samples in order of increasing impact velocity: 73, 100 and 122 m/s, respectively. In the dynamically deformed samples, a decrease in grain size is observed with the formation of grains less than 1 µm in size. Near the impact surface, we find a number of pore-like structures with a diameter of 10–40 µm (Figure 10a–c), and localization bands (Figure 8 and Figure 10b). The pore-like structures are not observed in the as-received material and, together with the grain refinement, are specific features of the further evolution of material microstructure during the dynamic deformation. The pore-like structures are indicated as grooves with defocused bottoms on the studied cross-sections of the deformed parts of the samples and, most likely, represent weak areas of the material crumbled during the etching. 

In the case of moderate impact velocities (up to 100 m/s) and reduced cylinders, small cracks are observed near the transition from the head part to the rest part; see Figure 8 for the case of a 4-mm reduced cylinder. In the case of a 3 mm cylinder at an impact velocity of 89.3 m/s, larger cracks are observed; the distance between the crack tips is 2.4 mm, with the original diameter of the reduced part being 3 mm. With an increase in the impact velocity of a 3-mm reduced cylinder to more than 100 m/s, a crack propagates throughout the entire transition diameter of the sample, as shown in Figure 9a,b and Figure 10a–c. In the case of a reduced 4 mm cylinder at an impact velocity of 120.5 m/s, the crack does not extend over the entire length of the transition diameter, and the distance between the cracks remains about 1 mm. In the present modeling, growth of cracks and fracture of material are not considered, but this experimental observation will be used in the further development and verification of fracture models. The formation of cracks was not observed for classical Taylor tests [23].

### 3.3. Bayesian Identification of Model Parameters

To describe experiments by the SPH method, model parameters for the material under consideration must be identified. We select part $\left\{\rho_{I}^{\delta }\left(t=0\right),\;A_{I},\;V_{I}\right\}$ of the model parameters to be fitted to our present experiments, while the rest are taken from the previous literature; see Table 1. These parameters were selected due to the following reasons: The initial density of immobilized dislocations $\rho_{I}^{\delta }\left(t=0\right)$ is directly related to the sample pre-processing and can be substantially different for the considered cold rolled rods. The hardening coefficient $A_{I}$ and the immobilization velocity $V_{I}$ determine the shear strength of the deformed material and, consequently, to the greatest extent, influence the final shape of the impacted samples. The rest of the model parameters characterizing the short-term plastic reaction of the material can be more efficiently determined from either analysis of the elastic precursor and plastic front of a plane shock wave at small strains [30,31], or fitted to MD simulations. The rate of dislocation immobilization ($V_{I}$) and the strength of dislocation structures ($A_{I}$) are long-term effects that cannot be captured in MD simulations. A similar situation exists for the initial state of material ($\rho_{I}^{\delta }\left(t=0\right)$). In future development of the model parameterization, a combination of short-term MD data with relatively long-term experiments is a prospective approach to optimizing the whole set of parameters.

**Figure 11:**
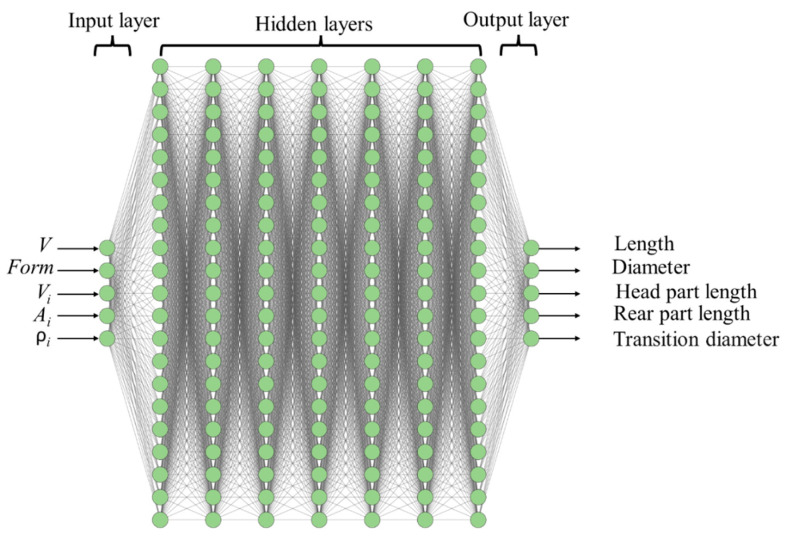
ANN structure for SPH model emulation: 7 × 21 hidden layers, 5 inputs, and 5 outputs; neurons are arranged in layers, and signals are transmitted in one direction—from the input layer to the output layer.

Taking into account different forms of samples and different impact velocities, a manual selection of model parameters requires too much time, and the choice of the best result by this method is not guaranteed. An artificial neural network (ANN) and Bayesian algorithm are used to automate the search for optimal parameters. The statistical Bayesian algorithm involves checking a lot of trial sets of parameters and requires a large number of calculations if implemented directly with the SPH. Therefore, the ANN is used as a fast emulator of the SPH model; the structure of the used ANN is presented in Figure 11.

**Figure 12:**
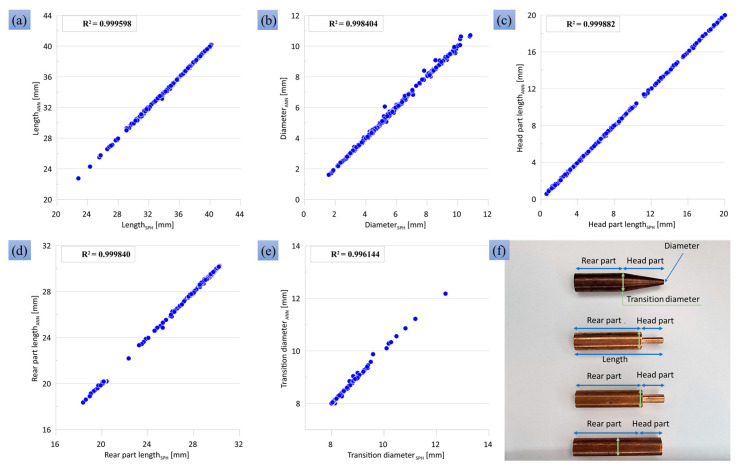
Correlation curves. Checking the accuracy of the neural network on validation data: (**a**) length ratio; (**b**) diameter ratio; (**c**) head part length ratio; (**d**) rear part length ratio; (**e**) transition diameter ratio; (**f**) scheme for measuring output parameters.

Using the SPH method, we prepare a database for training the ANN (more than 2000 results) and a validation database for checking the accuracy of the ANN training (350 results). The database is a set of the corresponding input and output values. The input of the ANN contains five values: $\left\{V_{x},\Psi ,\rho_{I}^{\delta }(t=0),A_{I},V_{I}\right\}$, where $V_{x}$ is the impact velocity, Ψ is a code of sample shape (0 for the uniform 8-mm cylinder, 1 for the reduced 4-mm cylinder, 2 for the reduced 3-mm cylinder and 3 for the truncated cone), $\rho_{I}^{\delta }\left(t=0\right)$ is the initial density of immobilized dislocations in each slip system, $A_{I}$ is the hardening coefficient, and $V_{I}$ is the immobilization rate. The output of the ANN characterizes the degree of deformation and the shape of the deformed sample after impact. It contains the following five values: the final length of the sample, the final diameter of the colliding end, the final length of the head part, the final length of the main (rear) part of the cylinder, and the transition diameter after impact. The output values were chosen in such a way as to take into account the shape of the samples under strong deformations. For example, in the case of a reduced cylinder at a high impact velocity, the main 8-mm part of the cylinder is deformed, and it must be taken into account for a good agreement between the experiment and the numerical calculation; this deformation is characterized by the transition diameter. The schema for measuring the output parameters is shown in Figure 12f.

The input parameters were played randomly with a uniform distribution within the ranges shown in Table 2. With these input parameters, the SPH was calculated. The process of calculating values for the database was parallelized using the OpenMP approach (version 3.1). It is used to carry out a large number of calculations of the same type; each thread performs its own calculation of a numerical experiment with its own set of parameters. Each numerical calculation was carried out until the rear part of the sample stopped, after which the thread received the next task with a new set of parameters.

A fully connected feed-forward artificial neural network is used. It consists of 5 neurons of the input layer, 5 neurons of the output layer, and 7 hidden layers with 21 neurons in each layer (see Figure 11). The number of neurons is chosen as $4N^{\mathrm{inputs}}+1=21$, where $N^{\mathrm{inputs}}$ is the number of input parameters, and previous experience showed that this rule is more efficient than the traditionally used $2N^{\mathrm{inputs}}+1$, especially for complex dependencies. The Sigmoid Linear Unit (SiLU) function is used as a transfer function for all neurons, and the cross-entropy loss function is used as a loss function. The training of an ANN comes down to finding the weights and biases of neurons that provide the minimum error when mapping input data to output data. The ANN was trained by backpropagation using batch gradient descent and the ADAM optimization algorithm. The ANN was trained using our own FORTRAN code; this was performed to ensure compatibility with the SPH code. The ANN training process was parallelized using the OpenMP approach. Each computing thread trains its own example of the ANN with a unique set of initial randomly seeded parameters of neurons, batch size, and training rate, which in aggregate lead to an individual learning trajectory for each example of the ANN; the best one is selected by the end. The number of hidden layers and the number of neurons in each hidden layer are constants for all computing threads and ANN examples. The ANN includes 3008 fitted parameters, which are the weights and biases of artificial neurons. The training database contains more than 2000 training examples of SPH simulations with 5 output parameters for each simulation, which means more than 10,000 numbers to be fitted in total. After the training process, a set of weights and biases for ANN neurons is selected that shows the highest accuracy. We managed to train ANN to the level of the root mean square error of 0.4% and the maximum error of 4.62% on the validation data; correlation curves are shown in Figure 12a–e. The quality of the ANN on the validation dataset evidences that there is no over-fitting. A decrease in the number of hidden layers leads to an increase in the maximum error above 5%; therefore, 7 hidden layers are used to ensure a maximum error below 5%. Control of the maximum error is important besides control of the root mean square error because an ANN with a high maximum error and a low root mean square error can give unpredictable output, although for a small number of points.

After training the ANN, it is used as a fast emulator of the SPH model to determine the optimal model parameters by means of the Bayesian algorithm of model calibration. From the experiment, we have a list of 50 rows containing impact velocity $V_{x}$, sample shape Ψ and five values to be compared (the final length of the sample, the final diameter of the colliding edge, the final length of the head part, the final length of the rear part of the cylinder, and the transition diameter). Three selected model parameters $\left\{\rho_{I}^{\delta }\left(t=0\right),\;A_{I},\;V_{I}\right\}$ are played out randomly within the ranges coinciding with those used for ANN training (see Table 2). For each particular set of model parameters $\left\{\rho_{I}^{\delta }\left(t=0\right),\;A_{I},\;V_{I}\right\}$, two other inputs of the ANN, $\left\{V_{x},\;\Psi \right\}$, run through all 50 experiments on the list. For each experiment, all five inputs are fed to the ANN that calculate the output values, and the probability of matching the output values with the experimental values is estimated using the equation:

(27) $$P=\exp\left\{-\sigma \sum_{n=1}^{N^{EXP}}\sum_{j=1}^{5}{\left(Y_{j,n}^{EXP}-Y_{j,n}^{ANN}\right)}^{2}\right\},$$

where σ is a normalization constant, $Y_{j,n}^{ANN}$ is the *j*-th output of the ANN for the *n*-th experiment, $Y_{j,n}^{EXP}$ are the corresponding experimental characteristics of the deformed sample, and $N^{EXP}=50$ is the number of different experiments (different sample shapes and impact velocities). The summation in Equation (27) is carried out over all output neurons. The higher the probability, the better the chosen parameters describe the experimental results. The search for optimal parameters using a fast emulator based on ANN and Bayesian approaches was also parallelized using OpenMP.

Both experimental and numerical data have certain errors, first of all due to the lack of exact axial symmetry. SPH results are obtained with a finite discretization, which is the case for any numerical method. In Equation (27), average values for both experimental and numerical data are compared; there is no special treatment of the error ranges in this estimation of the probability of the parameter set. On the other hand, the Bayesian approach is a statistical method relying on a large number of model-experiment comparisons, and uncertainties in both numerical and experimental data are compensated by these multiple comparisons. In our case, the numerical model is compared with $5N^{EXP}=250$ experimental data, as shown in Equation (27).

**Figure 13:**
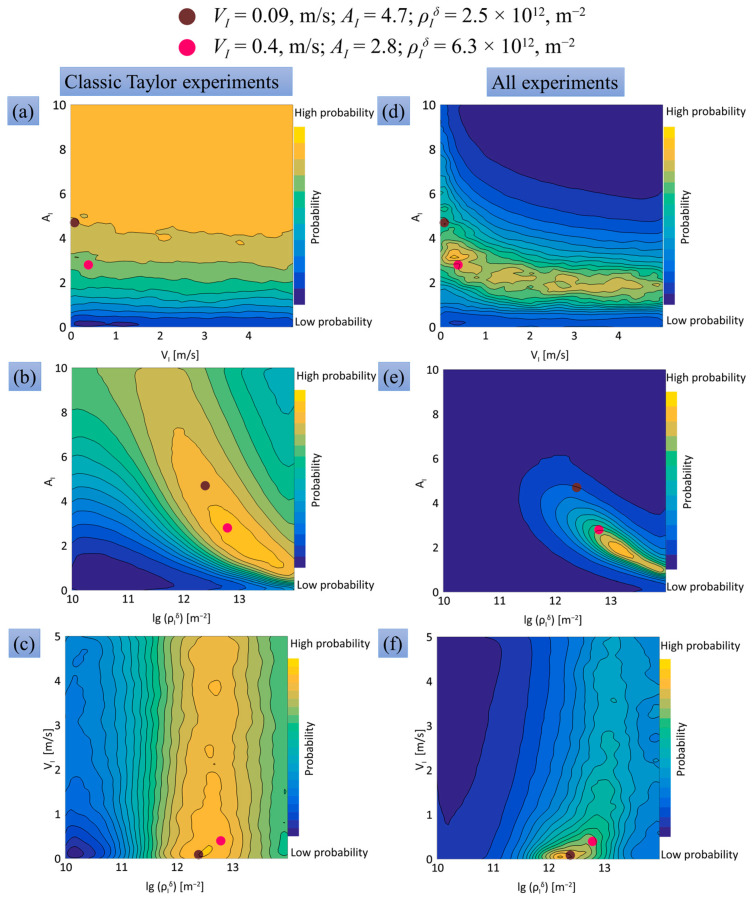
Probability distribution maps of model parameters: (**a**,**d**) $V_{I}-\;A_{I}$, (**b**,**e**) $\rho_{I}^{\delta }-\;A_{I}$, and (**c**,**f**) $\rho_{I}^{\delta }-\;V_{I}$. The distribution presented in (**a**–**c**) is plotted using only classical Taylor cylinders, while the distribution presented in (**d**–**f**) is plotted using the whole set of experiments; 100,000 random draws are used to plot these distribution maps. Little circles show the two highest local maxima for the case of a probability map with 10 Billion draws and a whole set of experiments to be further analyzed.

In Figure 13d–f, probability distribution maps of the model parameters are constructed using 100,000 draws and the entire set of experiments for comparison. Other numbers of draws are also considered for the convergence study. At maximum, 10 billion draws are employed; building such maps using a fast ANN-based emulator took 7 h on an 8-core, 16-thread AMD Ryzen 2700 processor, while 100 calculations of the SPH model on the same processor in parallel mode took 1 week. Ten billion draws means only about 2000 values for parameters, but even such a grid is excessive and implemented mostly due to the fast operation of the ANN-based emulator. Previous implementations of the Bayesian approach showed that even 40–50 values per parameter are often enough to localize the region of optimal parameters. This point is also evident from Figure 13, which uses only 100,000 draws, which means about 46 values per parameter, but allows one to localize the areas of maximal probability. The probability maps show that the areas of high probability form relatively narrow stripes on the planes in parameter space. These stripes mean that the improper variation of one parameter can be partially compensated by two other parameters. For instance, an increase in the immobilization velocity $V_{I}$ or the initial density of immobilized dislocations $\rho_{I}^{\delta }\left(t=0\right)$ can be partially compensated by a decrease in the hardening coefficient $A_{I}$ (see Figure 13a,b). On the other hand, preferable values of the immobilization velocity $V_{I}$ and the initial density of immobilized dislocations $\rho_{I}^{\delta }\left(t=0\right)$ can grow simultaneously (see Figure 13c). According to the dislocation plasticity model, an increase in either the initial density of immobilized dislocations or the immobilization velocity increases the current density of immobilized dislocations, and a simultaneous decrease in the hardening coefficient returns the static yield stress in Equation (20) to a level corresponding to the experimental data. This can explain the reverse dependence between the high-probability values of $V_{I}$ and $\rho_{I}^{\delta }\left(t=0\right)$, on the one hand, and $A_{I}$, on the other hand.

**Figure 14:**
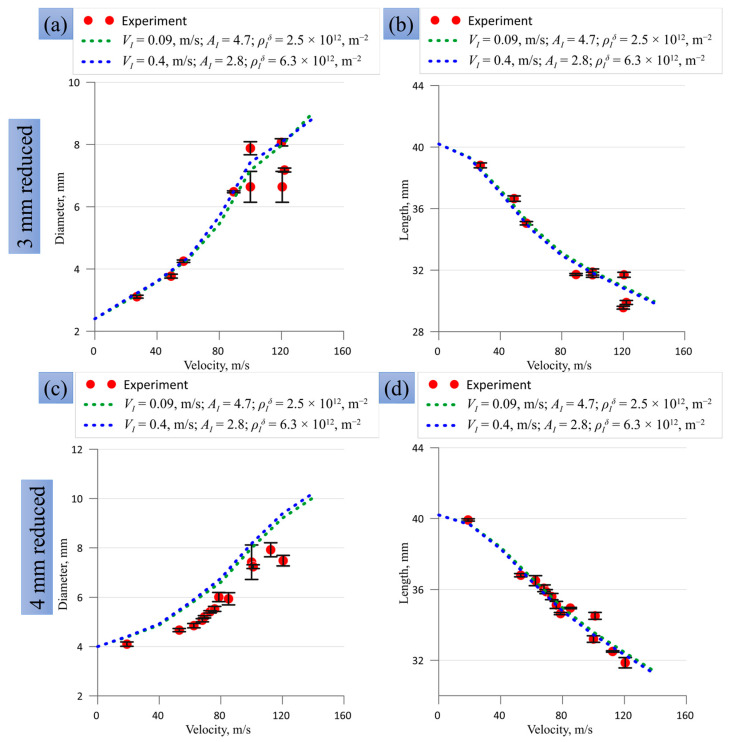
Comparison of numerical results (SPH) with the experimental data: Dependencies on impact velocity of (**a**) the final diameter of the impact edge and (**b**) the final length of a 3-mm reduced cylinder; (**c**) the final diameter of the impact edge and (**d**) the final length of a 4-mm reduced cylinder. Green lines show the first maximum, and blue lines show the second maximum.

**Figure 15:**
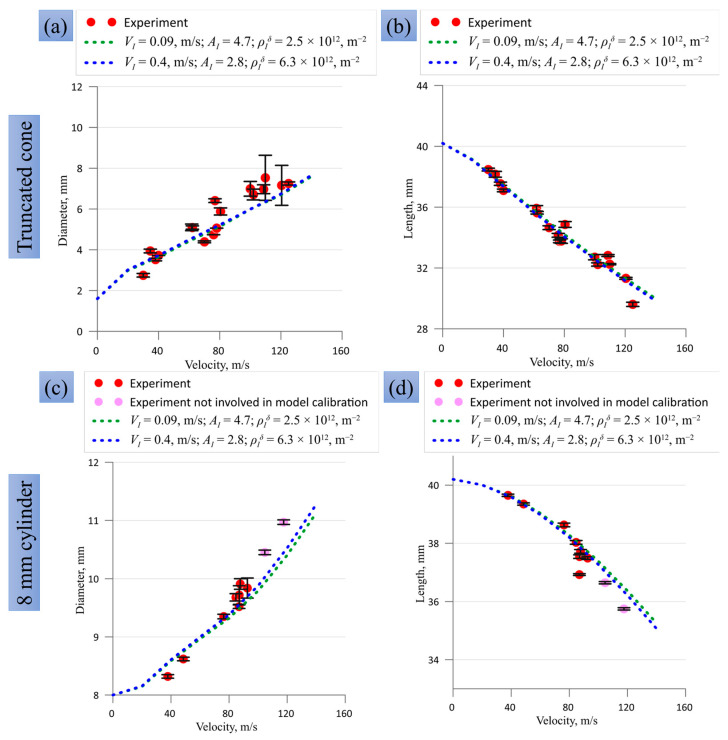
Comparison of numerical results (SPH) with the experimental data: Dependencies on impact velocity of (**a**) the final diameter of the impact edge and (**b**) the final length in the case of a truncated cone; (**c**) the final diameter of the impact edge; and (**d**) the final length of a uniform 8-mm cylinder. Green lines show the first maximum, and blue lines show the second maximum. Experimental points for the two highest impact velocities in (**c**,**d**) were not used in parameter calibration.

**Figure A1:**
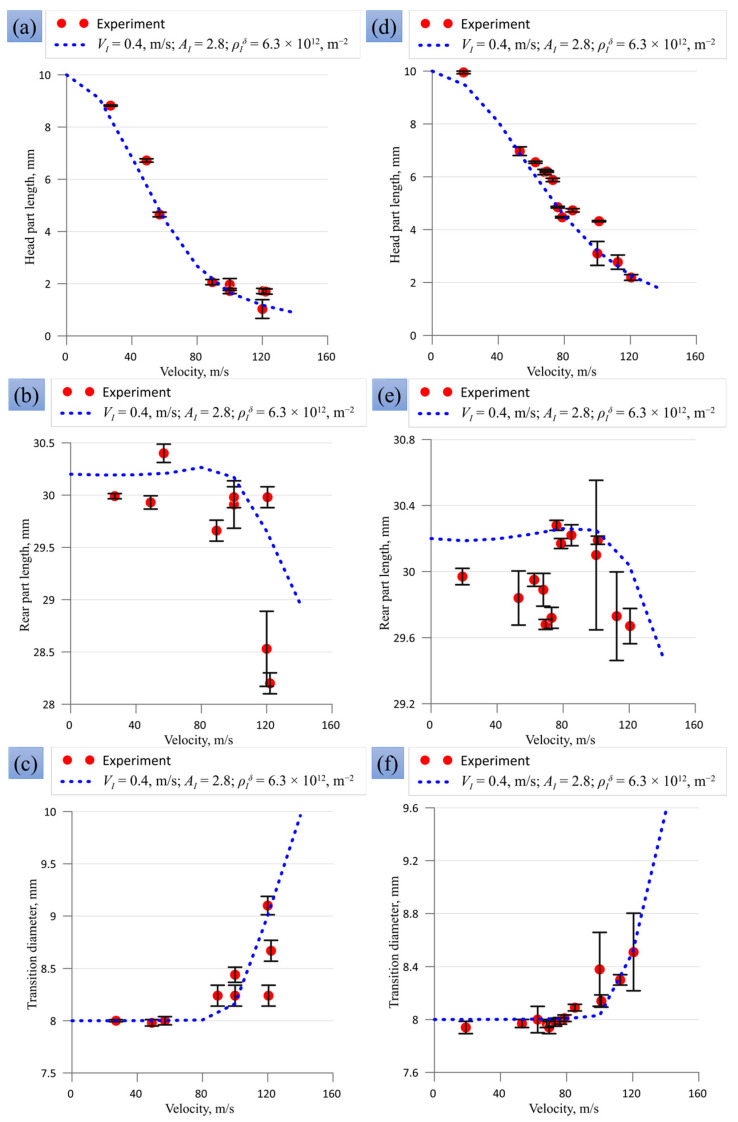
Comparison of numerical results (SPH) with the experimental data: dependence of the impact velocity on (**a**) the length of the head of the projectile for the case of a 3 mm reduced cylinder, (**b**) the length of the rear of the projectile for the case of a 3 mm reduced cylinder, (**c**) the transitional diameter of the projectile for the case of a 3 mm reduced cylinder, (**d**) the length of the head of the projectile for the case of a 4 mm reduced cylinder, (**e**) the length of the rear of the projectile for the case of a 4 mm reduced cylinder, (**f**) the transitional diameter of the projectile for the case of a 4 mm reduced cylinder.

**Figure A2:**
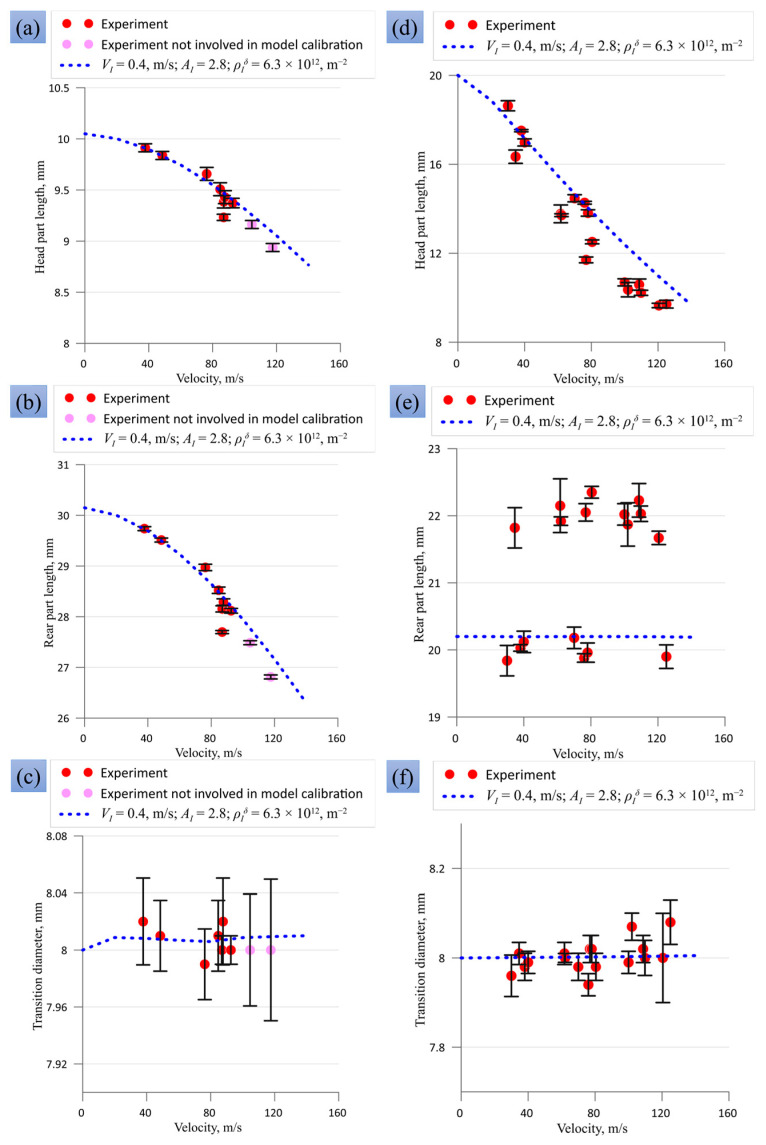
Comparison of numerical results (SPH) with experimental data: dependence of the impact velocity on (**a**) the length of the head of the projectile for the case of an 8 mm cylinder, (**b**) the length of the rear of the projectile for the case of an 8 mm cylinder, (**c**) the transitional diameter of the projectile for the case of an 8 mm cylinder, (**d**) the length of the head of the projectile for the case of a truncated cone, (**e**) the length of the rear of the projectile for the case of a truncated cone, and (**f**) the transitional diameter of the projectile for the case of a truncated cone.

In Figure 13, there are several areas with the highest probabilities in the presented two-parameter plots, which are projections of three-parameter space. These plots are shown for visualization purposes, while the model parameters with maximal probability can be found during the operation of the program algorithm for Bayesian probability calculation. Based on 10 billion draws, we consider the two highest local maxima with the following model parameters: (i) $V_{I}$ = 0.09 m/s, $A_{I}$ = 4.7, and $\rho_{I}^{\delta }$ = 2.5 × 10^12^ m^−2^, and (ii) $V_{I}$ = 0.4 m/s, and $A_{I}$ = 2.8 and $\rho_{I}^{\delta }$ = 6.3 × 10^12^ m^−2^. These two sets of parameters were used in the SPH calculations; a comparison with the experiment is shown in Figure 14 and Figure 15 for the final diameters of the impact edge and the final lengths, and in Figure A1 and Figure A2 of Appendix A for other compared sizes. Both maxima provide a close fit to the curves and a reasonable agreement with the experiment. The maximum number (ii) was chosen for further numerical calculations of SPH because it is closer to the model parameters previously used for modeling plane shock waves in [31]. One should note that using model parameters lying far from the area of maximal probability provides a much worse correspondence of the SPH-based model and the experiment.

Probability maps constructed using only the classical Taylor cylinders are plotted for comparison in Figure 13a–c. One can see that classical Taylor cylinders give a lower performance of model parameterization compared to the entire series of experiments: the high-probability zones are much wider and less determined; the optimal hardening coefficient is dismissed to higher values in Figure 13a, while the immobilization rate is not well-defined in Figure 13a,c. Therefore, the dislocation plasticity model parameterized in [23] with classical Taylor cylinders failed to describe the profiled samples, which motivated the present research. This shortcoming of the classical cylinders can be partially overcome by the experimental study of higher impact velocities to increase the strain rate and strain value, but using profiled cylinders more easily solves this problem.

**Figure A3:**
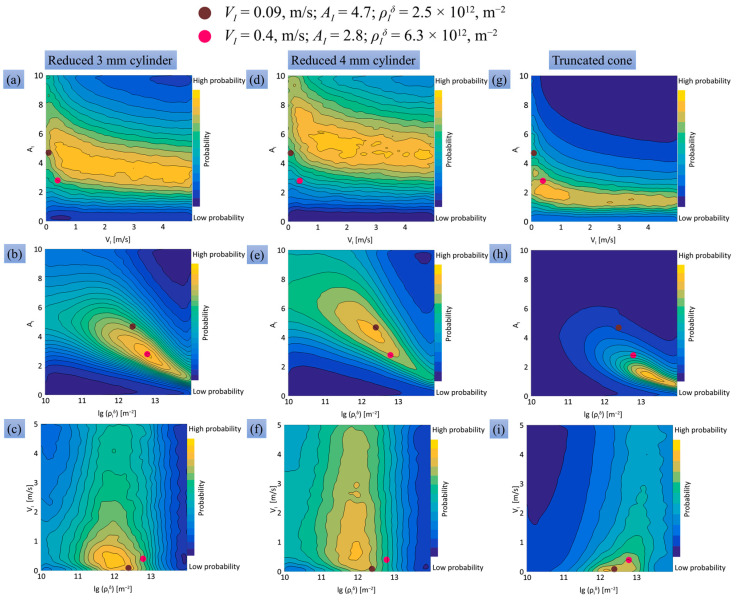
Probability distribution maps of model parameters: (**a**,**d**,**g**) $V_{I}-\;A_{I}$, (**b**,**e**,**h**) $\rho_{I}^{\delta }-\;A_{I}$, and (**c**,**f**,**i**) $\rho_{I}^{\delta }-\;V_{I}$. The distributions presented in (**a**–**c**), (**d**–**f**) and (**g**–**i**) are plotted using only 3-mm reduced cylinders, 4-mm reduced cylinders, and cones in the head part, respectively; 100,000 random draws are used to plot these maps. Little circles show the two highest local maxima for the case of a probability map with 10 Billion draws and a whole set of experiments.

In order to further elucidate the performance of different subsets of profiled cylinders for model parameterization, similar probability maps for cases of only 3-mm reduced head parts, 4-mm reduced head parts, and cone-shaped head parts are plotted and collected in Figure A3 of Appendix B. Each one of these subsets is more efficient than the classical cylinders because they give narrower zones of high probability of model parameters. The highest performance is for the cone head part, Figure A3g–i, followed by 3-mm cylinders, Figure A3a–c, and the lowest performance for 4-mm cylinders, Figure A3g–i. The relatively low performance of 4-mm cylinders is explained by the about 3 times lower strain rate achieved and the about 2 times lower true strains achieved compared with the case of truncated cones. At the same time, each subset gives a displaced estimation of the model parameters, which means that they are better optimized for this particular problem, but worse at reproducing an entire series of experiments. Therefore, using multiple shapes is beneficial.

**Figure A4:**
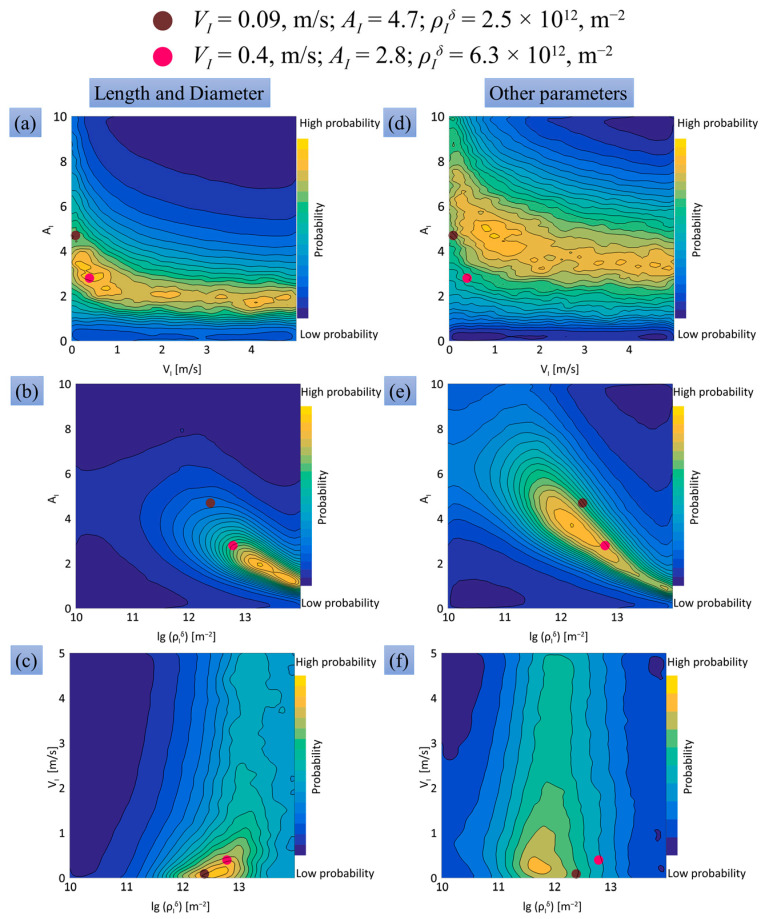
Probability distribution maps of model parameters: (**a**,**d**) $V_{I}-A_{I}$, (**b**,**e**) $\rho_{I}^{\delta }-\;A_{I}$ and (**c**,**f**) $\rho_{I}^{\delta }-\;V_{I}$. The distribution presented in (**a**–**c**) is plotted using only the total final length and diameter of the impact spot, while (**d**–**f**) is plotted using only three other parameters (head part length, rear part length, and transition diameter) instead. Little circles show the two highest local maxima for the case of a probability map with 10 Billion draws and a whole set of experiments.

For model parameterization, we use five characteristics of the deformed samples: the total final length, the diameter of the impact spot, the head part length, the rear part length, and the transition diameter. This set of characteristics is selected to describe in compact form the most essential features of the sample deformation. For instance, the rear part length and the transition diameter deviate from their initial values only at severe deformations of profiled samples at high impact velocities. The probability maps accounting for only the total final length and the diameter of the impact spot are presented in Figure A4a–c, while the case of using the remaining three parameters instead is presented in Figure A4d–f. A comparison with Figure 13d–f shows that the first couple of characteristics makes the main contribution and can be used separately. The remaining three characteristics are auxiliary, and using only these characteristics gives a displaced estimation of the model parameters.

Let us consider the coincidence between the optimized SPH and the experiments presented in Figure 14, Figure 15, Figure A1 and Figure A2 (Appendix A) in more detail. The SPH gives a statistically reasonable description of both trends and values of the final length and diameter of the impact edge for all sample shapes. The coincidence is not absolute, but the optimization process varies only three model parameters to describe 50 experiments with different sample shapes simultaneously. The deviation for large impact velocities does not exceed the scatter and uncertainties of the experimental data. Experimental points for the two highest impact velocities in Figure 15c,d for classical Taylor cylinders were not used in parameter calibration, but they are adequately reproduced by the model. The SPH itself as a numerical method is well-suited for large deformations, but the present version of the dislocation plasticity model is formulated in terms of small deformations. Reformulation of the dislocation plasticity model using the formalism of finite strains can increase the precision of the numerical model at the highest impact velocities and will be tested in future work. The evolution of the head part length (Figure A1a,d and Figure A2a,d) to a certain extent repeats the evolution of the total length (Figure 14b,d and Figure 15b,d). The rear part length and the transition diameter start to change only at high impact velocities in the case of 3-mm and 4-mm head parts (Figure A1b,c,e,f), indicating strong plastic deformation of the head part. In the case of the conic head part, these two sizes remain unchanged in the examined range of impact velocities (Figure A2e,f); this means that the plastic deformation is completely localized in the cone head part. In the case of classical cylinders, all auxiliary sizes vary in the entire range of impact velocity (Figure A2a–c).

### 3.4. Result of SPH Modeling in Comparison with the Experiment

**Figure 16:**
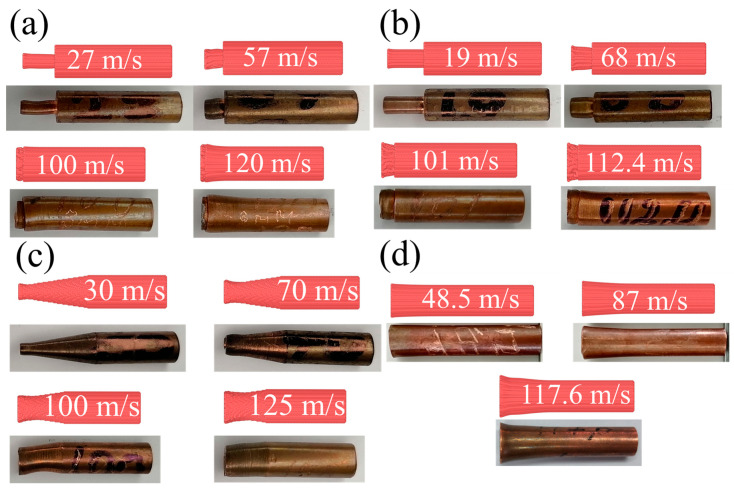
Comparison of the final shape of the sample after deformation for different shapes of impactors: (**a**) reduced 3-mm cylinders; (**b**) reduced 4-mm cylinders; (**c**) truncated cones; (**d**) uniform 8-mm cylinders.

Here, we present the results of SPH modeling with the optimal parameters identified in the previous section. The purpose of this analysis is both to compare the model results with the experiments and to reveal the deformation conditions of the samples using the combined experimental-numerical approach. A comparison of the final shape of the experimental samples with the numerical model is shown in Figure 16 for all examined shapes of impactors and for several selected impact velocities. One can see a good qualitative correspondence between the model and the experiment.

#### 3.4.1. SPH Results for the Uniform 8-mm Cylinder

Taking into account the optimized parameters, it makes sense to recalculate and compare the previously published [23] results for a uniform 8-mm cylinder (classic Taylor test). A comparison of the calculated sample shape with the experiment is presented in Figure 16d and shows a typical “mushrooming” of cylinders with an increase in impact velocity.

**Figure 17:**
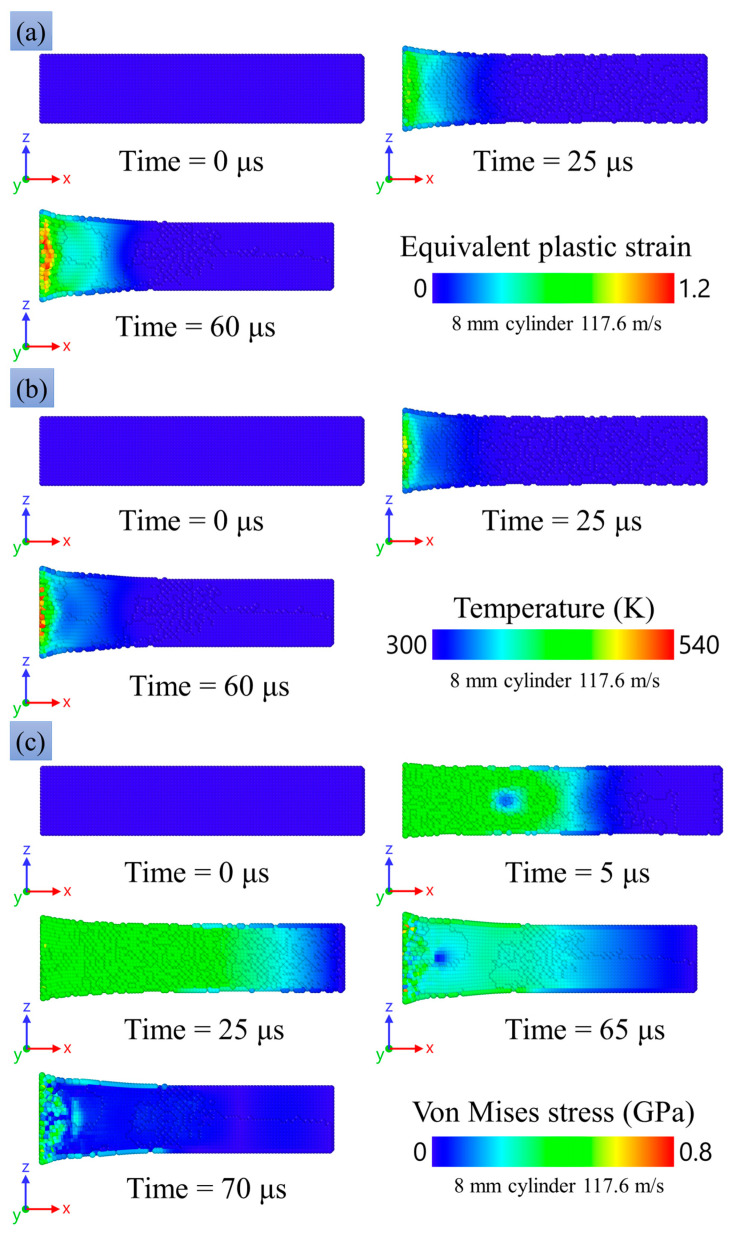
SPH calculations for a uniform 8-mm cylinder impacted at a velocity of 117.6 m/s: Spatial distributions of (**a**) the equivalent plastic strain, (**b**) the temperature, and (**c**) the von Mises equivalent stress in the central cross-section in subsequent moments of time.

Figure 17a shows the equivalent plastic strain $w_{M}$, which is a scalar measure of the plastic strain tensor w:

(28) $$w_{M}=\sqrt{\left(2/3\right)\left(w:w\right)}.$$

The plastic deformation is concentrated near the impact surface; as it propagates towards the middle part of the sample, the plastic deformation decays down to zero. In the center of the contact surface, the values of plastic deformation are maximum, which was also already confirmed by microphotographs of the deformed experimental sample in [23]. In the experimental sample, the largest number of pores was observed in the regions of the greatest plastic deformations. It evidences agreement between the numerical experiment and the microstructural analysis of the samples.

Figure 17b shows the temperature distribution in the numerical experiment. The highest temperatures are obtained in the central part of the impact surface of the striker. The maximum temperature increase is about 160 K.

Figure 17c shows the spatial distribution of the von Mises equivalent stress $\sigma^{M}$ which is calculated through the stress deviator S, as follows:

(29) $$\sigma_{M}=\sqrt{\left(3/2\right)\left(S:S\right)}.$$

and is equal to the flow stress in the case of plastic flow at simple tension or compression. After the onset of deformation, one can see a uniform distribution of stresses almost along half of the sample, with stresses ranging approximately 0.35–0.45 GPa. The highest stresses are observed on the impact surface. After stopping the sample and further rebound, a sharp drop in the stresses is observed. 

**Figure 18:**
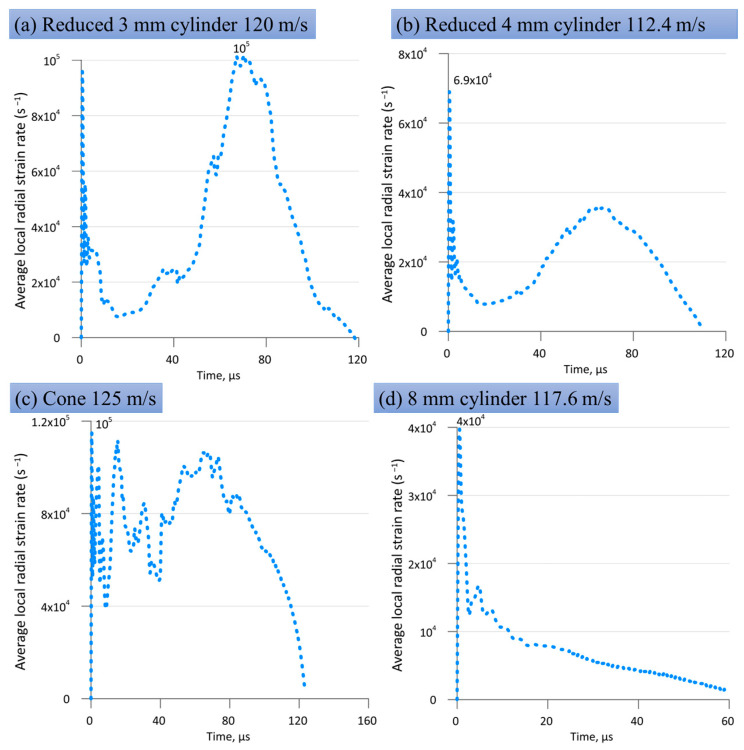
Temporal evolution of the radial strain rate for: (**a**) a reduced 3-mm cylinder impacted at 120 m/s; (**b**) a reduced 4-mm cylinder impacted at 112.4 m/s; (**c**) a truncated cone impacted at 125 m/s; (**d**) a uniform 8-mm cylinder impacted at 117.6 m/s.

Figure 18d shows the time evolution of the radial strain rate at the impact edge of a sample that hits an obstacle at a velocity of 117.6 m/s. The data were obtained by numerical calculation. At the beginning of deformation, the strain rate reaches a peak value of 4 × 10^4^ s^−1^, then decreases with small oscillations until the sample stops; the stopping time is about 70 μs.

#### 3.4.2. SPH Results for Reduced Cylinders

Figure 16a,b compares SPH modeling and the experimental result for the final shapes of the impacted copper specimens with the head part in the form: (a) a 3-mm reduced cylinder, and (b) a 4-mm reduced cylinder. The modeling repeats the experimental tendency of shortening of the head part, with the almost undeformed remaining part acting as a hammer. The degree of shortening increases with the increase in impact velocity, similar to both the experiment and the modeling. The radial expansion is close to uniform along the head part, while some tendency to the formation of a barrel-shaped profile is observed, similar to the experiment. A bending of the head part is observed in some of the experimental photos, while there is no bending in the present modeling. On the other hand, we observed bending in preliminary modeling with less balanced numerical models. One can conclude that there are conditions for instability in the form of bending, and a small misalignment or disturbance of mass balance can initiate the instability. At high impact velocities (more than 100 m/s) for the cases of reduced 3-mm and 4-mm cylinders, deformation of the main 8-mm part of the cylinder is observed both in the experimental studies and in the numerical modeling.

**Figure 19:**
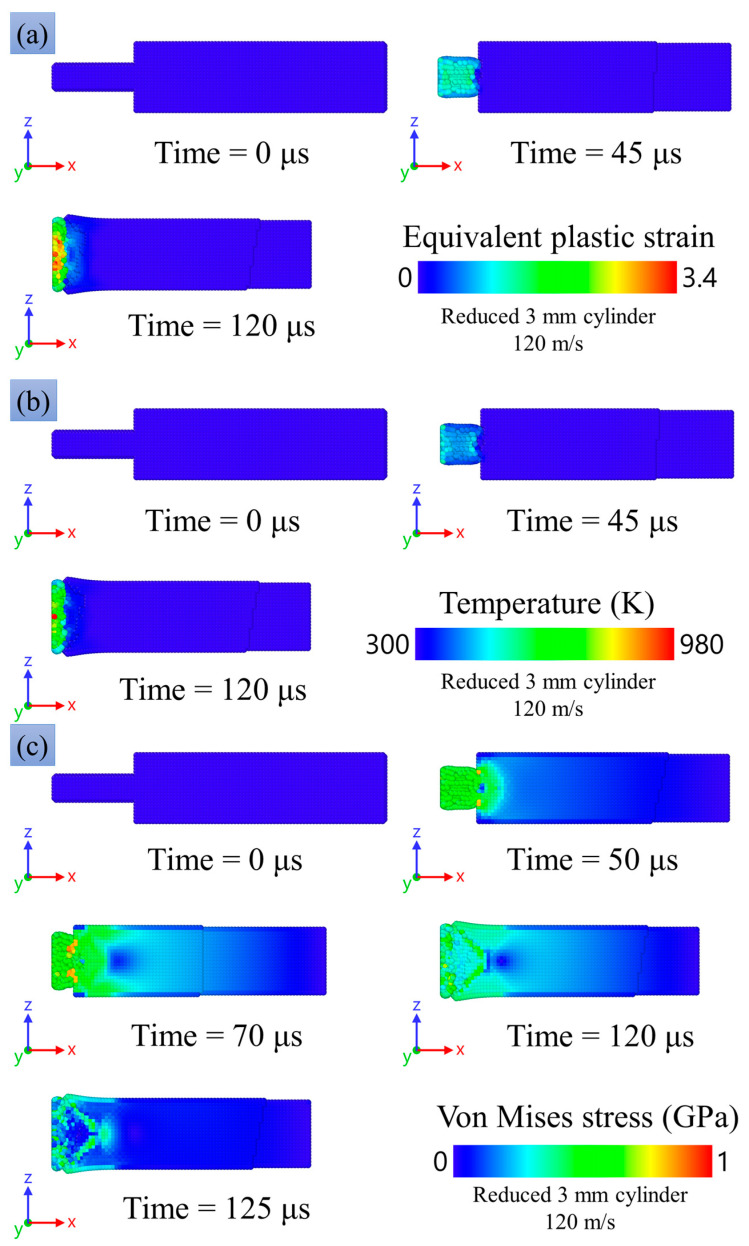
SPH calculations for a 3-mm reduced cylinder impacted at a velocity of 120 m/s: Spatial distributions of (**a**) the equivalent plastic strain, (**b**) the temperature, and (**c**) the von Mises equivalent stress in the central cross-section in subsequent moments of time.

**Figure 20:**
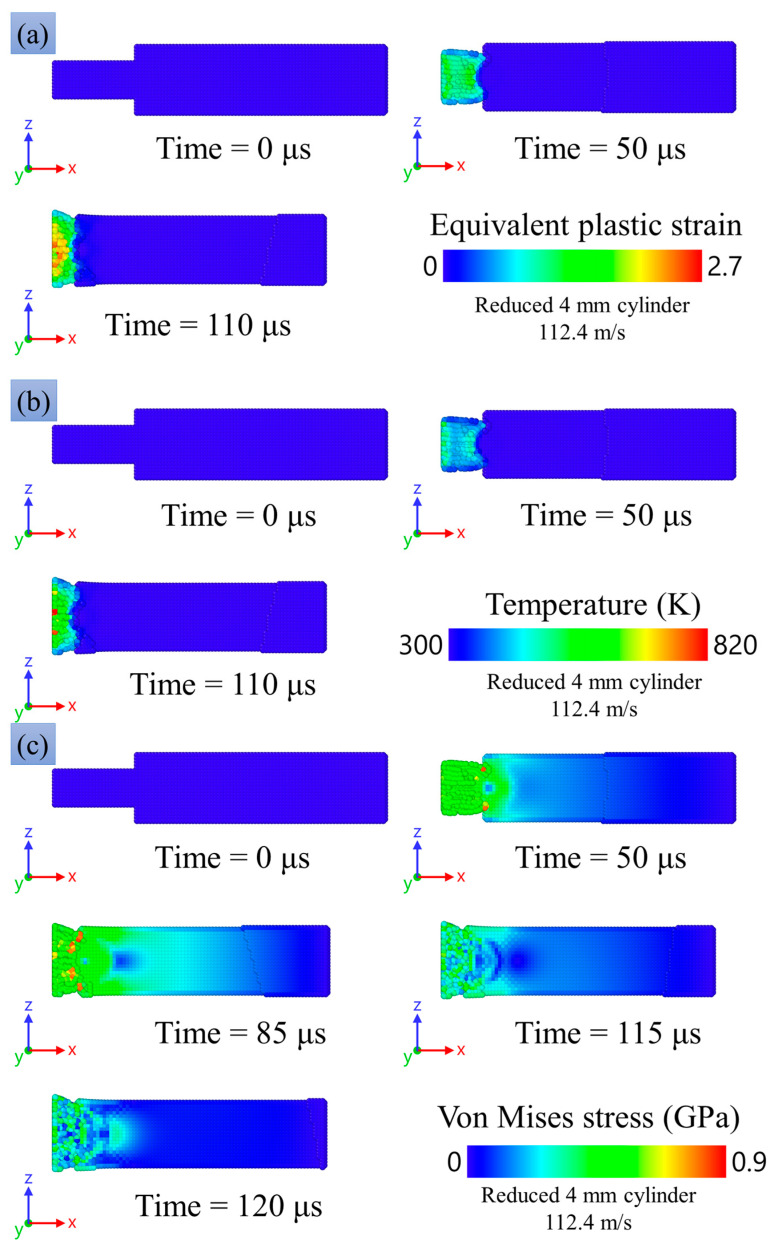
SPH calculations for a 4-mm reduced cylinder impacted at a velocity of 112.4 m/s: Spatial distributions of (**a**) the equivalent plastic strain, (**b**) the temperature, and (**c**) the von Mises equivalent stress in the central cross-section in subsequent moments of time.

Figure 19 and Figure 20 show the equivalent plastic strain, the temperature distribution, and the spatial distribution of the von Mises equivalent stress learned from the numerical modeling. Plastic deformation is mainly concentrated in the region of the reduced cylinder, while the values of plastic deformation in the case of a reduced 3-mm cylinder are higher due to the smaller cross-section area and, accordingly, larger stresses during deformation. The temperature distribution is similar to the plastic deformation distribution. The maximum temperature values are 980 K and 820 K for reduced 3-mm and 4-mm cylinders, respectively; the average temperature in the deformed part of the sample is about 650–700 K.

The von Mises stress in the samples reaches a value of about 0.9 GPa in the case of a reduced 3- and 4-mm cylinder. It can be seen in Figure 19c and Figure 20c at the time of 50 μs that the stress is maximum in the transition region of the reduced 3- and 4-mm cylinders to the 8-mm cylinder part. This is in good agreement with the results of microstructural analysis, which show small cracks in Figure 8 and large cracks up to through ones in Figure 9. Thus, one can speak of a good agreement between the numerical experiment and the results of the microstructural analysis of the samples.

Figure 18a shows the radial strain rate at the impact edge of a reduced 3 mm cylinder that hits an obstacle at a speed of 120 m/s. The data were obtained by numerical calculation. At the beginning of deformation, the deformation rate reaches a value of 9.6 × 10^4^ s^−1^, and then the deformation slows down. Thereafter, the main 8-mm cylinder begins to press on the reduced 3-mm cylinder, and the strain rate sharply increases to a peak value of 1 × 10^5^ s^−1^, then monotonously decreases before stopping the sample; the stop time is approximately 120 µs.

A similar situation is observed for a reduced 4-mm cylinder (see Figure 18b). At the beginning of deformation, the strain rate reaches a peak value of 6.9 × 10^4^ s^−1^. At the moment of pressure by the main 8-mm cylinder on a reduced 4-mm cylinder, the strain rate reaches values of 3.8 × 10^4^ s^−1^, then it monotonously decreases before stopping the sample; the stopping time is approximately 110 µs.

#### 3.4.3. SPH Results for Truncated Cones in the Head Part

**Figure 21:**
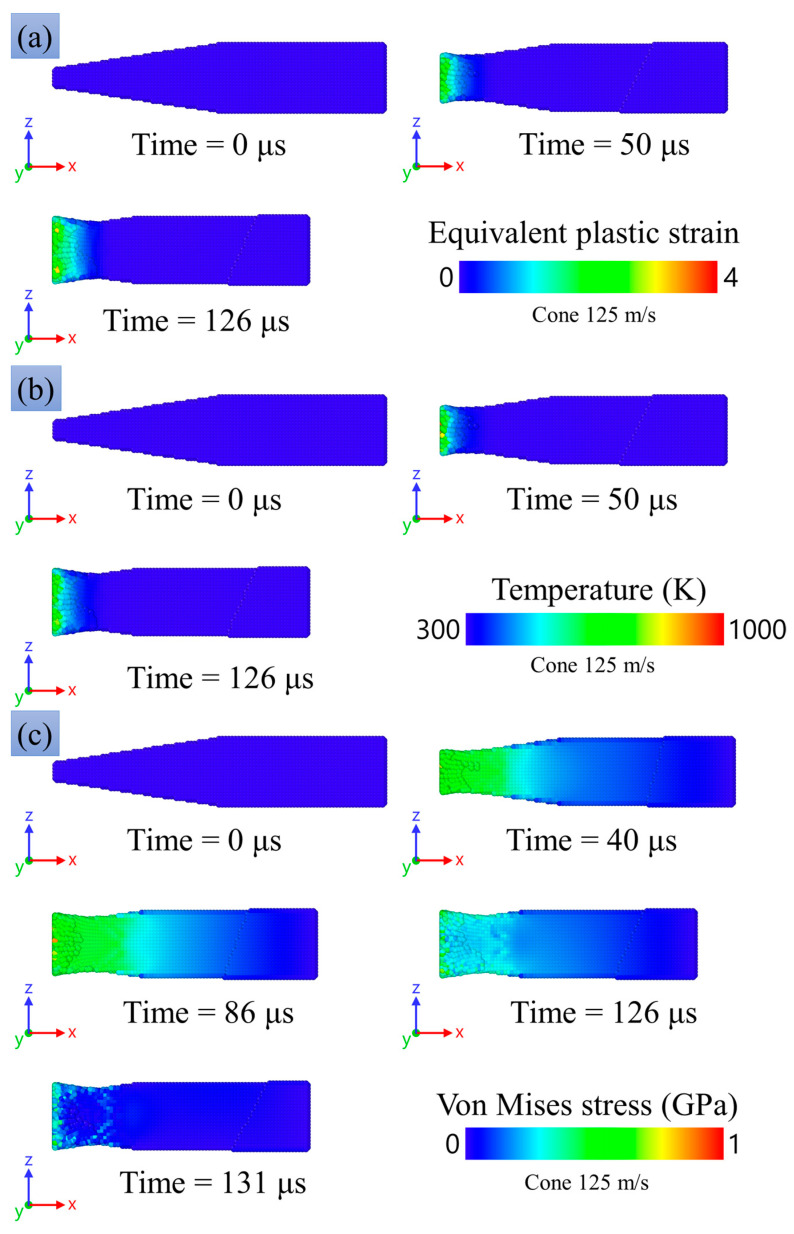
SPH calculations for a cylinder with a truncated cone in the head part impacted at a velocity of 125 m/s. Spatial distributions of (**a**) the equivalent plastic strain, (**b**) the temperature, and (**c**) the von Mises equivalent stress in the central cross-section in subsequent moments of time.

A comparison of the shapes of the deformed samples is presented in Figure 16c and shows the predominant flattening of the cone part. Figure 21a shows the distribution of plastic strain at different times. Plastic deformation is present mainly in the area of the contact surface. The temperature distribution is shown in Figure 21b. The temperature in the deformed region of a truncated cone averages 650–750 K, and the maximum temperature is about 1000 K, which is still far from the melting point of copper. The stress field distribution is shown in Figure 21c. The maximum stress on the contact surface is about 1 GPa, and the average for the head part of the sample is about 0.7 GPa.

Figure 18c shows the radial strain rate at the impact edge of a truncated cone impinging on an obstacle at a speed of 125 m/s. At the beginning of deformation, the strain rate reaches a peak value of 1 × 10^5^ s^−1^, and then oscillates at a level of about 0.8 × 10^5^ s^−1^ almost until stopping. The stopping time is approximately 123 µs, which coincides with the cases of reduced cylinders in the head part (Figure 21a,b) and about twice as much as in the case of a uniform 8-mm cylinder (Figure 21d).

### 3.5. Strain Rates during Impact

Figure 18 compares the calculated radial strain rates for all sample shapes reached at the maximal impact velocities of the corresponding samples. These strain rates are calculated from the change rate of the average radius divided by the current average radius of the contact spot between the sample and anvil from SPH modeling. The radial deformation of this contact spot is chosen for analysis as the most extreme deformation process during impact. The maximal strain rate varies from about 4 × 10^4^ s^−1^ for the classical Taylor cylinder in Figure 18d to about 10^5^ s^−1^ for the reduced 3-mm cylinder and truncated cone in Figure 18a,c. The highest strain rates are close to those observed in flyer plate experiments, but the achieved strains are orders of magnitude higher (the radial true strain reaches a value of about one, as one can estimate from Figure 14 and Figure 15). Thus, the proposed profiled samples make it easy to obtain experimental information about simultaneous large deformations and high strain rates. The profiling of cylinders increases the stresses and strains in the head part.

### 3.6. Estimation of Grain Refinement and Weakened Areas (Pore-like Structures)

**Figure 22:**
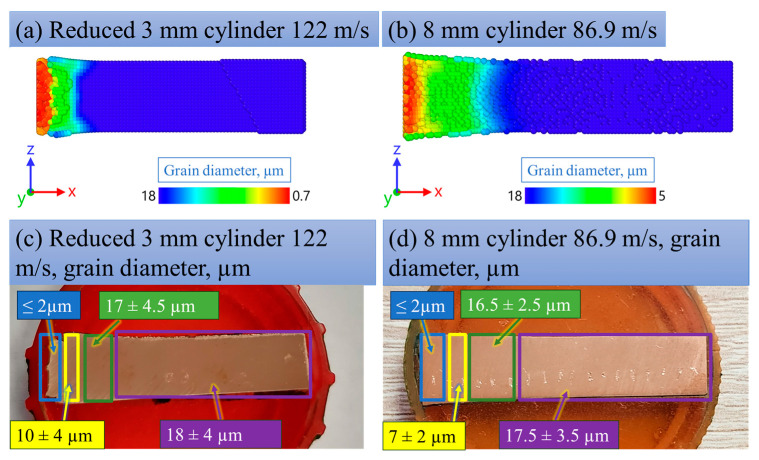
Calculated grain diameter in a Cu impactor for (**a**) a reduced 3 mm cylinder at an impact velocity of 122 m/s, and (**b**) an 8 mm cylinder at an impact velocity of 86.9 m/s.

**Figure 23:**
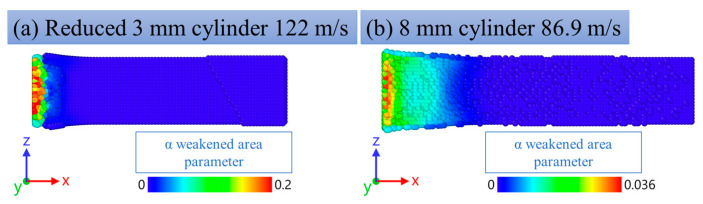
Calculated volume fraction of weakened areas (pore-like structures) in a Cu impactor for (**a**) a reduced 3 mm cylinder at an impact velocity of 122 m/s, and (**b**) an 8 mm cylinder at an impact velocity of 86.9 m/s.

Here, we numerically calculate the spatial distributions of the grain diameter and the volume fraction of weakened areas (pore-like structures) in the sample after deformation using an example of a reduced 3-mm cylinder impacted at 122 m/s (Figure 22a and Figure 23a) and a classical 8-mm cylinder impacted at 86.9 m/s (Figure 22b and Figure 23b). The former sample was subjected to optical microscopy in the present study (Section 3.2), while the latter sample was in the previous one [23]; therefore, one can compare calculation results with the experimental data. The numerical calculation is based on Equations (22) and (24) supplementing the dislocation plasticity model.

In the case of a reduced cylinder, the numerical simulation predicts a strong grain refinement with the grain diameter decreasing down to about 0.7 µm in the deformed head part (Figure 22a). This agrees well with the results of microstructural analysis shown in Figure 22c: the grain size near the impact surface is less than 2 µm, which is the threshold of the used optical microscopy. The thickness of the zone with refined grains also coincides between the experiment (Figure 22c) and modeling (Figure 22a). The initial diameter $d_{0}=d\left(t=0\right)\approx 18\;\mu m$ is taken from the experimental data for the as-received material. In the case of an 8 mm sample at an impact velocity of 86.9 m/s, the minimum grain size in the deformed region is 5 µm in the numerical calculation (Figure 22b). For classical cylinders, [23] found a stronger refinement near the impact plane with a prevailing number of grains with a size less than 2 µm, but with the retention of a part of coarse grains with a size of 17 µm close to the initial one. At the same time, conglomerates of grains with an average size of 7 µm prevail at a distance of 3 mm from the impact plane [23], which is in line with our numerical results; compare Figure 22b,d. In general, our numerical estimates for grain refinement do not contradict the experimental data.

As for the volume fraction ***α*** of weakened areas (pore-like structures), one can expect correspondence in the order of magnitude. According to our numerical simulations with Equation (24), the maximal volume fraction is 35% (of the order of tens of percents) for the case of a 3-mm reduced cylinder, and it is 3.6% (of the order of several percents) for the case of a classical 8-mm cylinder. The experimental value ranges from 10% to 53% in the former case and is about 7.5% in the latter case, which correlates with the numerical predictions, at least in order of magnitude. The experimental values were estimated by using optical microscopic images of 0.5 mm × 0.5 mm areas of etched cross-sections with calculation of the areal fraction of pores and approximate recalculation into the volume fraction. For the experimental analysis, we took into account all pore-like structures and localization bands, but tried to avoid zones with cracks. For a reduced 3 mm cylinder, the experimental *α* was calculated both near the impact plane and in the region of the transition from the reduced 3-mm cylinder to the main 8-mm part between cracks. In the case of the classical 8-mm cylinder, we examined only the region near the impact surface, since as one moves away from it, the number of weakened zones decreases due to a smaller amount of plastic deformation similar to that presented in Figure 23b.

### 3.7. Comparison with Flyer Plate Experiments

**Figure 24:**
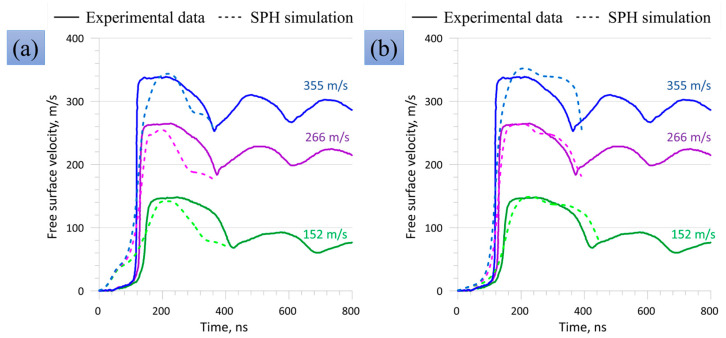
Comparison of SPH modeling with the experimental data by Kanel et al. [108] for high-velocity plate impact. Free surface velocity histories for impact velocities of 152, 266, and 355 m/s with experimental data are presented by solid lines, and the calculation data are presented by dashed lines. Calculation with the parameters collected in Table 1 (in particular, $\rho_{I}^{\delta }\left(t=0\right)=6.3\times 10^{12}\;m^{-2}$ in each slip system) is shown in panel (**a**), while calculation with a reduced initial density of immobilized dislocations of $\rho_{I}^{\delta }\left(t=0\right)=10^{11}\;m^{-2}$ is shown in panel (**b**). Calculation data are clipped by the time moment of the first spall pulse in experiments because the spall fracture is not considered in the present SPH modeling.

The parameterized model is compared with the results of flyer plate experiments by [108] at room temperature. In these experiments, the impacted samples were made of OFHC copper of M1 grade, but annealed in vacuum at 445 °C for 20 min; therefore, the initial dislocation density is expected to be much lower than in our cold-rolled samples. The copper samples had a thickness of about 2.47 mm and were impacted by a copper flyer plate with a thickness of 0.56 mm, which accelerated to a velocity in the range of 150–355 m/s. In the experiment, the perpendicular dimensions of the plates ensure uniaxially deformed states (plane shock waves) in the region of interest in the middle of the plates during the registration period. In order to obtain similar conditions in our 3D SPH modeling, the interacting system is represented by a highly elongated parallelepiped of 5 × 6 × 500 SPH particles, with the long side along the impact direction, which prohibits motion of SPH points in perpendicular directions. Figure 24 compares our calculated free surface velocity histories with the experimental data [108] for three impact velocities. As one can expect, calculations with a high initial dislocation density of $\rho_{I}^{\delta }\left(t=0\right)=6.3\times 10^{12}\;m^{-2}$ in each slip system (about $0.8\times 10^{14}\;m^{-2}$ in total on 12 slip systems) optimized for our cold-rolled samples overestimate both the elastic precursor and elastic release (the first velocity drop behind the maximum) wave, as shown in Figure 24a. At the same time, reducing this parameter down to $\rho_{I}^{\delta }\left(t=0\right)=10^{11}\;m^{-2}$ in each slip system ($1.2\times 10^{12}m^{-2}$ in total) while retaining all other model parameters gives a reasonable correspondence between SPH modeling and experiments for the shape of the shock front and release wave, as shown in Figure 24b. The parameter $\rho_{I}^{\delta }(t=0)$ characterizes the initial deformed state of material; therefore, it must be different between the pre-deformed and annealed samples. It should be noted that the value of the dislocation density as low as $10^{11}\;m^{-2}$ can be observed in annealed materials [107], while the value of the order of $10^{14}\;m^{-2}$ is typical for deformed materials.

## 4. Discussion

Traditionally, experiments with the simplest possible loading conditions are used in dynamic testing of materials to simplify analysis of the obtained results and extract the required parameters, such as dynamic yield strength and spall strength. The modern development of numerical simulations and machine learning approaches allows one to apply more complex loadings for parameter identification. On the one hand, it increases tolerance for the quality of the experiments performed. On the other hand, it expands the spectrum of examined deformed states and makes the identified model more consistent with real conditions in engineering practice. Following this logic, we develop a combined experimental-numerical approach both to identify the model parameters and to study the dynamic plasticity of metals. This approach is applied to the case of cold-rolled OFHC copper.

In the experimental part, profiled samples with reduced or conical head parts are proposed for the Taylor impact problem for the first time for material characterization. These samples make it possible to achieve large plastic deformations with strain rates up to 10^5^ s^−^^1^ at impact velocities of less than 130 m/s, which is close to the strain rates typical for the impact of metal plates of millimeter thickness. The profiled head part of the samples experiences the most severe plastic deformation. Microstructural analysis shows substantial refinement of grains and the formation of pore-like structures in the deformed head parts of the samples. High impact velocities lead to the development of cracks in the transition zone from the head part to the main part of the impacted samples. 

The experimental results are used for optimization of parameters of the dislocation plasticity model [31], implemented in a 3D case with the numerical scheme of SPH [23]. Applying the statistical Bayesian method and a trained ANN as an SPH emulator, parameters of the dislocation plasticity model are optimized to obtain the best fit with the results of experiments for all sample shapes. The sensitivity of the optimization algorithm to different subsets of training data is studied. It is shown that classical Taylor cylinders are not enough for a univocal selection of the model parameters, while the profiled cylinders provide better optimization even if used separately. The combination of different shapes and an increase in the number of experiments increases the quality of optimization.

It is shown that the results of the SPH calculation with the optimized model are in statistically reasonable correspondence with experiments for the final shape of the impacted samples. The results of the numerical modeling, including the spatial distributions of stress and plastic strain, are in agreement with the areas of concentration of pores and large cracks determined from the metallographic analysis of the experimental samples. The optimized initial value of dislocation density $0.8\times 10^{14}\;m^{-2}$ is relatively high, but corresponds to the estimations based on optical metallography. SPH calculations show a further increase in the density of immobilized dislocations in the head part, while the density of mobile ones remains about two orders of magnitude lower and even decreases due to immobilization. The increase in dislocation density is saturated due to annihilation processes. According to the supplemented dislocation plasticity model, the energy released in the dislocation annihilation is further spent on the grain refinement and formation of weakened areas of material revealed as pore-like structures in the optical microscopy. And this assumption allows one to describe qualitatively and even quantitatively the experimental observations in the formation of such structures: the areas of modification and the estimated values coincide between numerical simulations and experiments. This addition to the dislocation plasticity model opens a way for the description of various plasticity-induced microstructures and the development of the fracture model with explicit accounting of preceding plasticity processes.

The optimized numerical model is successfully used for description of the shock wave profiles registered in the recent flyer plate experiments by Kanel et al. [108]. The obtained correspondence validates the dislocation plasticity model, its numerical SPH implementation and the developed procedure of parameter optimization.

In total, we propose a cheap, simple, but efficient route for optimizing the parameters of a dynamic plasticity model. Model parameters specify the material and its initial state; therefore, optimization is necessary for each material separately. Besides copper, the proposed approach is successfully applied in the case of brass (not included in the present paper). Possible practical applications of the optimization procedure include laser short peening [109], which requires a reliable material model to numerically investigate the shock-wave-induced residual stresses. Further development of this work supposes reformulation of the dislocation plasticity model in the formalism of finite deformations and the combination of experimental data and MD simulations for optimization of the model parameters.

## Figures and Tables

**Table 1:** Parameters of the dislocation plasticity model for copper. Most of the parameters are taken from [31]. The dislocation friction coefficient is based on MD simulations [80,81]. “Optimized” in the Reference column means that parameters are fitted to the present experiments by means of the Bayesian statistical method (Section 2.5).

Parameter	Value	Reference
B [Pa·s]	$0.45\times 10^{-5}+2.5\times 10^{-8}\times T$	[80,81]
μ	0.34	[31]
$Y_{s0}$ [MPa]	30	[31]
$A_{I}$	2.8	Optimized
$V_{I}$ [m/s]	0.4	Optimized
$k_{D}$ [J^−1^]	7.8 × 10^16^	[31]
$k_{A}$	5	[31]
$\rho_{D}^{\mathrm{free}}$ [m^−2^]	10^11^	[31]
$\rho_{D}^{\delta }\left(t=0\right)$ [m^−2^]	10^11^	[31]
$\rho_{I}^{\delta }\left(t=0\right)$ [m^−2^]	6.3 × 10^12^	Optimized

**Table 2:** Ranges of ANN input values.

Parameter	Value
$V_{x}$ [m/s]	0–150
Ψ	0–3
$\log_{10}\left(\rho_{I}^{\delta }\right)$ [m^−2^]	10–15
$A_{I}$	0–10
$V_{I}$ [m/s]	0–5

**Figure A5:**
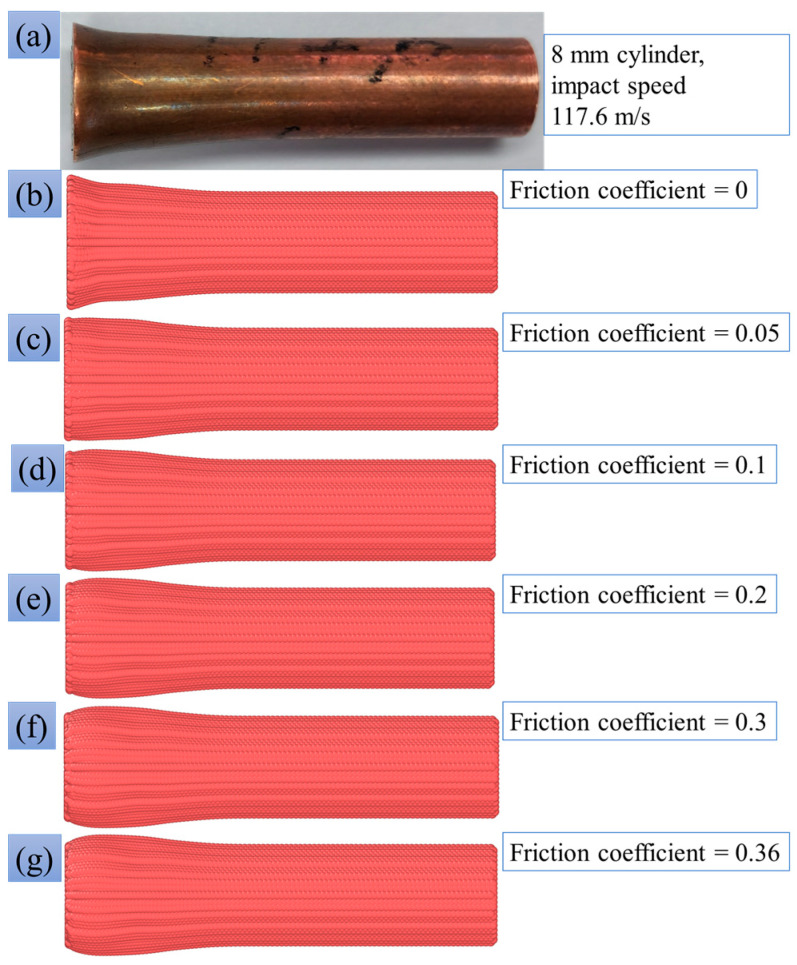
Comparison of the experimental shape with the results of numerical simulation at different friction coefficients for an 8 mm cylinder with an impact velocity of 117.6 m/s: (**a**) photograph of the sample shape after impact; (**b**–**g**) shapes of numerical samples with friction coefficients of 0, 0.05, 0.1, 0.2, 0.3, and 0.36, respectively.

**Figure A6:**
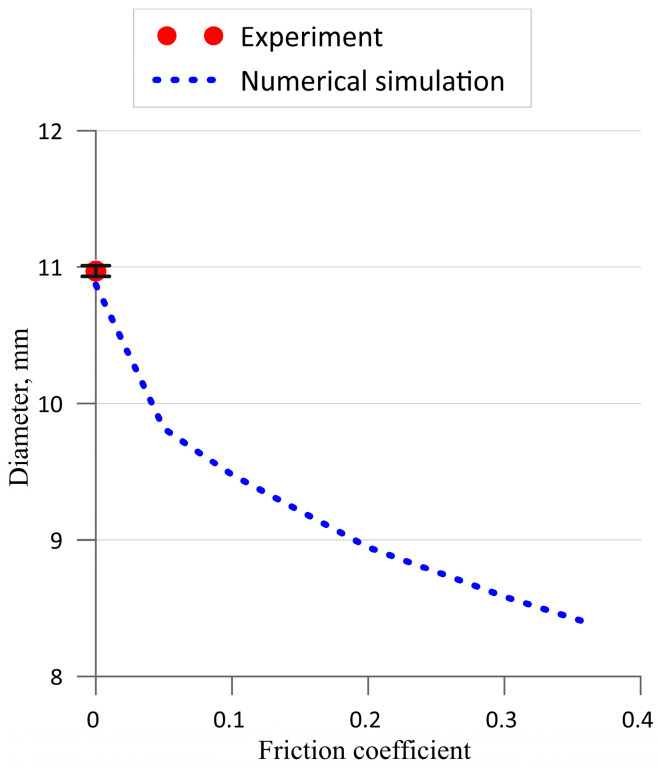
Dependence of the impact surface diameter on the coefficient of friction for an 8 mm cylinder, impact velocity of 117.6 m/s. Red dot—experimental sample; blue dotted line—numerical experiment.

## Data Availability

The most essential data of the experiments and modeling are presented in the paper. Other data are available upon request.

## Appendix A. Comparison of Auxiliary Sizes between SPH and Experiment

To better match the shape of the numerical sample with the experimental one, it is proposed to characterize the dimensions of the samples in the numerical calculation and experiment as shown in Figure 12f. In the main text, the total length and the diameter of the impact spot are considered, while here the results for the three remaining auxiliary sizes are collected. Comparisons of experimental and numerical dimensions are shown: for the case of a 3-mm reduced cylinder in Figure A1a–c; for the case of a 4-mm reduced cylinder in Figure A1d–f, for the case of an 8 mm classical cylinder in Figure A2a–c; and for the truncated cones in Figure A2d–f. The SPH with optimized model parameters gives a reasonable description of the change in the length of the head and rear parts and the transition diameter for all sample shapes. In the case of cones, Figure A2e, we have experimental data for two series of samples with the initial rear part length differing by about 2 mm. Both series are used because this difference in the initial shape does not substantially influence the taper angle and deformation during impact. In this case, the optimized SPH does not show a change in the final length of the rear part of the cone-shaped samples with an increase in the impact velocity, and this behavior is similar to that observed in the experiment.

## Appendix B. Parametric Study for the Bayesian Algorithm: Training with Different Parts of Data

The probability maps constructed with partial experimental data are collected here in order to show the sensitivity of the Bayesian parameterization to these different parts of the entire experimental dataset. The accounting of only 3-mm reduced cylinders, 4-mm reduced cylinders and cones in the head part is shown in Figure A3a–c, Figure A3d–f, and Figure A3g–i, respectively, while the case of only classical Taylor cylinders is shown in Figure 13a–c. The case of accounting for only the total final length and the diameter of the impact spot is shown in Figure A4a–c, while the case of using the remaining three parameters instead is shown in Figure A4d–f.

## Appendix C. Effect of Friction on the Final Shape of the Sample

The influence of friction between the sample and anvil on the deformed shape is examined here. Figure A5 compares the shape of the experimental sample with a numerical experiment with different friction coefficients in the case of an 8 mm cylinder at an impact velocity of 117.6 m/s. Figure A6 shows the dependence of the final diameter of the impacted edge on the friction coefficient for numerical calculations; for convenience, the graph also shows the final diameter of the experimental sample. The best match of the shape and diameter of the sample after impact is obtained with zero friction; reparameterization is not an option because the shape of the modeling with friction qualitatively differs from the experiment. An increase in the friction coefficient leads to a decrease in the diameter after impact and significantly affects the shape: the formation of a material overflow near the impact surface is observed in the numerical experiment, while on the impact surface itself, the diameter of the sample changes slightly, which contradicts the experimental shape. This is explained by the fact that in the experiments, the samples were lubricated with WD-40 to reduce friction during acceleration and impact.
